# Evaluating Methods for High-Dimensional Mediation in Metabolomics Data

**DOI:** 10.1021/acs.est.5c09706

**Published:** 2026-01-07

**Authors:** Susan S. Hoffman, Donghai Liang, Anne Dunlop, Todd Everson, Audrey J. Gaskins, Dean P. Jones, Anke Hüls, Michele Marcus, Ashley I Naimi

**Affiliations:** † Department of Epidemiology, 1371 Emory University, Atlanta, Georgia 30322, United States; ‡ Gangarosa Department of Environmental Health, 1371 Emory University, Atlanta, Georgia 30322, United States; § Department of Gynecology and Obstetrics, School of Medicine, 1371 Emory University, Atlanta, Georgia 30322, United States; ∥ School of Medicine, 1371 Emory University, Atlanta, Georgia 30322, United States

**Keywords:** metabolomics, simulation, high-dimensional mediation, meet-in-the-middle, HIMA, HDMA

## Abstract

This study evaluated high-dimensional mediation analysis methods (HIMA by Zheng et al. and HDMA by Gao et al.) and the “Meet-in-the-Middle” (MITM) approach using simulated metabolomics data. Simulations varied in sample size, mediator set size, correlation structure, proportion of true mediators, and mediation effect size (beta). We assessed each method’s ability to estimate the total indirect effect (TIE), component indirect effects (CIEs), sensitivity, and specificity. In scenarios with independent metabolites, HIMA and HDMA reliably estimated CIEs, while HDMA provided the most accurate estimate of the TIE. MITM generally underestimated the TIE, and HIMA showed improved TIE estimates with higher mediator effect sizes. In correlated settings, CIE estimation was not feasible due to the lack of identifiable causal contrasts, and all methods underestimated the TIE. Sensitivity declined in low beta, small sample size, and high-dimensional scenarios, though specificity remained high (>90%) across all methods. Findings suggest that HIMA offers the most accurate mediation results but may exclude meaningful features through dimensionality reduction. Therefore, applying parallel mediation approaches, such as MITM and HIMA, and focusing on the overlapping findings would be recommended. These results underscore the need for the development of robust, scalable mediation methods tailored to untargeted metabolomics data.

## Graphical abstract

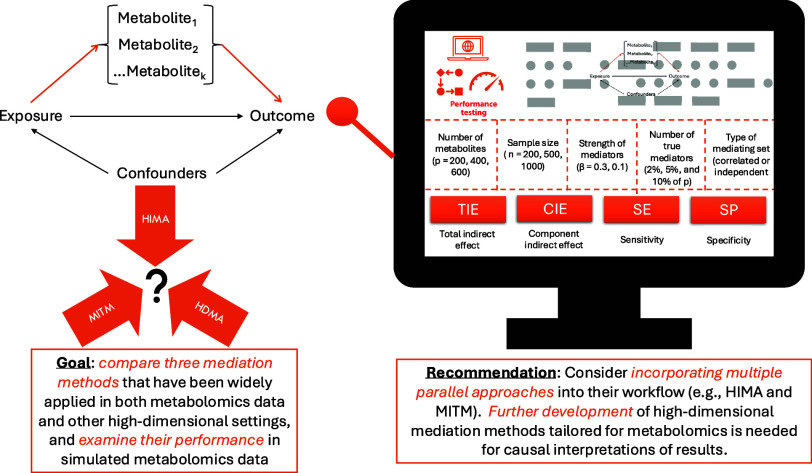

## Introduction

The metabolome, the set of small-molecule chemicals found within a cell, tissue, or organism, provides a biologically functional measurement of gene-environment interactions and gene transcription. While metabolomics has emerged as a sensitive analytical platform that helps to describe the biological underpinnings mediating exposure–outcome relationships,^1,2^ several recent papers have highlighted that existing approaches often fail to adequately account for mediator-to-mediator correlation, scale poorly when the number of mediators far exceeds the sample size, and may yield conservative or biased estimates.^3–5^ Improving this understanding would help elucidate the complex relationship among exposure, metabolome, and disease. Examples of high-dimensional techniques include mediation analysis methods initially developed for other fields like epigenetics (HIMA^6^ and HDMA^7^), as well as the meet-in-the-middle (MITM)^8^ approach.

Per- and polyfluorinated substances (PFAS) have been linked to many biological perturbations, including amino acids, fatty acids, glycerophospholipids, glycerolipids, phosphosphingolipids, bile acids, ceramides, purines, and acylcarnitines.^9^ High-dimensional mediation methods have been used to identify which metabolites and metabolic pathways may underlie the connection between exposure to environmental pollutants, such as PFAS, and adverse health outcomes, including fetal growth impairment.^10–12^ These initial investigations highlight the feasibility and potential of high-dimensional mediation in metabolomics. For example, prenatal PFAS exposure has been associated with altered levels of branched-chain amino acids,^9^ which may contribute to impaired fetal growth through perturbation of amino acid and energy metabolism. Mediation analysis provides a framework to formally test such pathways, allowing researchers to assess whether changes in branched-chain amino acids or other metabolite classes statistically mediate the relationship between PFAS exposure and birth outcomes. Quantifying the mediation effect statistically provides insight into the proportion of the total exposure–outcome relationship explained by specific metabolic pathways, offering a clearer understanding of biological mechanisms and potential intervention targets.

A mediator is a variable that is influenced by the exposure and impacts the outcome along the causal pathway.^13^ Classic causal mediation, such as first described by Baron and Kenny,^14^ provides a framework for identifying variables that mediate the association between an exposure and outcome. This can be described through the direct and indirect effects of exposure on an outcome. The indirect effect refers to the effect of an exposure on an outcome that is due to some third intermediary variable (the mediator), while the direct effect refers to the effect of an exposure on an outcome that does not depend on the intermediary. When data are used to estimate these effects, the assumptions required for interpreting direct and indirect effect estimates causally are fairly strong. One key assumption is conditional exchangeability, which requires no uncontrolled confounding between (1) the exposure and the mediator, (2) the exposure and the outcome, and (3) the mediator and the outcome. An additional fourth assumption required when using classical causal mediation methods is that no mediator-outcome confounder can be affected by the exposure.^15^ If this fourth assumption is violated, more sophisticated techniques based on the suite of “g methods” must be used.^16,17^ Otherwise, several problems arise, including the potential collider stratification bias.^18^

Unfortunately, classic mediation methods cannot be applied to high-dimensional mediation settings due to the complex mutual influences of biological processes and the thousands of identified features per participant inherent in metabolomic data. For example, a recent study examining the impact of polychlorinated biphenyls (PCBs) on the metabolome identified 21 metabolites within the same biochemical cascade.^19^ Many identified metabolites exhibit strong interdependence; for instance, 4-aminobutanate (GABA) is a direct precursor to glutamic acid. If one were to hypothetically link these metabolites to a health outcome using existing high-dimensional mediation methods, the causal assumptions required by those methods would be violated. Specifically, these approaches require that mediators act independently and without confounding, yet in reality, it is almost impossible to assume that a given mediator (e.g., GABA) has no mediator–outcome confounder (e.g., glutamic acid) that is also affected by the exposure. Appropriate statistical methods for examining high-dimensional mediation have only begun to be explored. Among the mediation methods available to researchers, some likely perform better than others when dealing with complex metabolomics data. Thus, identifying and exploring different mediation options and evaluating their performance in metabolomic data would improve our understanding of which methods to use.

Simulation studies play a crucial role in statistical research and methodology development by applying computer-generated data that closely resemble real-world data. These studies provide a valuable tool for researchers to assess the suitability of statistical methods for addressing specific research questions within the context of specific data. Simulation studies are more important in the era of complex high-throughput data and the application of advanced machine-learning algorithms. By creating synthetic data sets with known characteristics, researchers can systematically evaluate the performance of different methods under various conditions.^20^ This enables researchers to assess how well the methods can uncover the simulated “truth” or estimand, which is unattainable in real-world data where the true values are unknown. Such insights into method behavior and performance allow researchers to identify the strengths and limitations of different approaches, improving methodology and preventing potential reproducibility issues in future studies.^21^ Simulation studies provide researchers with a controlled environment to explore, refine, and validate statistical methods, enhancing the reliability and robustness of data analyses in real-data applications.

Currently, there is no standard method for conducting mediation analyses in untargeted metabolomics data. Many complicating factors include high dimensionality, a mediating set often larger than the sample size, and complex biological interactions. The goal of this research was to address this critical gap by comparing three mediation methods that have been applied in both metabolomics data and other high-dimensional settings and to examine their performance in simulated metabolomics data. These methods include the HIMA,^6^ HDMA,^7^ and MITM^8^ approaches.

## Methods

A simulation study was conducted to improve the understanding of how high-dimensional mediation analysis methods perform in metabolomics data. This research used simulated data based on the metabolomic characteristics observed in the Atlanta African American Maternal-Child Cohort (Atlanta AA cohort),^10,22–25^ to understand how changes in this high-dimensional mediator impact the ability of various methods to estimate the total indirect effect (TIE) through all metabolic features, the component indirect effect (CIE) through individual features, and the sensitivity and specificity of the captured metabolites. The TIE represents the portion of the total effect mediated by or explained by the intermediate variables. The TIE is part of the total effect, which encompasses both the direct effect (the effect of the exposure on the outcome that is not mediated by the intermediate variables) and the indirect effect (the effect of the exposure on the outcome that operates through the intermediate variables). The CIE allows for understanding how much a single feature contributes to the total indirect effect (Figure S1). Researchers can understand the overall causal relationship among the exposure, intermediate variables, and outcome by decomposing the total effect into its direct and indirect components.

### High-Dimensional Mediation Analysis

Three high-dimensional mediation analysis approaches, HIMA,^6^ HDMA,^7^ and MITM,^8^ were evaluated in this study. These methods do not account for mediator–outcome confounding that is affected by the exposure,^16^ which is a key consideration when applied to real-world metabolomics data, where complex feedback loops and exposure-induced confounding are common and can bias estimates of the mediation effect.

1. HIMA is a penalization-based regression method adopted from the multiple mediator model’s framework and extends this methodology to a high-dimensional setting (i.e., where the number of mediators is larger than the sample size).^6,26^ HIMA has demonstrated effectiveness in epigenetic data but has not been formally assessed in metabolomic data.^6^ HIMA has output for both the TIE and the CIE. Briefly, HIMA follows three general steps: (1) the pool of potential mediating factors is reduced to a number that is less than the number of individuals in the sample size, (2) variable selection is conducted using the minimax concave penalty (MCP),^27^ and (3) joint significance testing is conducted for mediating effects. In step 1, to reduce the number of mediators to a more manageable number, sure independence screening (SIS)^28^ is applied to the mediator-outcome model to select mediators with the largest effect on the outcome variable. Briefly, SIS is a variable selection technique that reduces the dimensionality of high-dimensional data by ranking covariates according to their marginal associations with the outcome. Of note, the mediators will be standardized to ensure that the coefficients represent the same scale. This step reduces the number of mediators to a value controlled by the researcher but less than the sample size. In step 2, the selected candidate mediators are modeled in the mediator-outcome model, relying on MCP to shrink mediator-outcome effects toward zero. Zheng et al. selected MCP as the preferred shrinkage method as it conducts variable selection in a computationally efficient, accurate, and unbiased manner compared to other methods (e.g., Least Absolute Shrinkage and Selection Operator [Lasso] in HDMA).^27^ In step 3, HIMA uses maximized *p*-values to evaluate the significance of the mediation effect and uses Bonferroni corrections to adjust results for multiple testing. The CIE for each selected mediator is calculated as the product of the exposure-to-mediator coefficient and the mediator-to-outcome coefficient, and the TIE is the summation of the CIEs from the selected mediators. We used the default implementation of HIMA in the R package hima.
2. HDMA is a high-dimensional mediation technique built on desparsified Lasso estimators.^7^ HDMA has output for both the TIE and the CIE. First, an SIS^28^ procedure is applied to lower the dimensionality of the mediating set to be less than the number of participants using the criteria: *d* = *n*/log (*n*), where *d* is the mediating set and *n* is the sample size. Next, the penalized regression model is applied. This model introduces a penalty term, effectively shrinking the coefficients of the less important variables in the mediating set to 0. The goal of the penalized linear outcome model is to find the set of predictor variables with the strongest association with the outcome variable while simultaneously minimizing the impact of irrelevant or weakly associated variables. To counteract any bias that may have been introduced to the coefficient estimates, a debias procedure is applied to attempt to provide more accurate estimates. Gao et al. has determined that the HDMA method is more statistically robust and efficient than other options as it fits multiple mediators in a single regression step instead of testing the mediation effect one mediator at a time compared to other high-dimensional mediating options (e.g., HIMA). The CIE in HDMA is calculated in a similar way as in HIMA: as the product of the exposure-to-mediator and mediator-to-outcome coefficients for each selected mediator. The TIE is the summation of the selected CIEs. We used the default implementation of HDMA, which is available in the package hdmed.^12^
3. The MITM approach combines untargeted metabolomics profiling with targeted metabolomics analysis to uncover features associated with the exposure of interest and the outcome of interest.^8^ First, an independent metabolome-wide association study (MWAS) is conducted separately between the exposure and the metabolome and between the metabolome and the outcome. Overlapping features and metabolic pathways are identified and considered representative of the intersecting signals associated with the exposure and the outcome. A simplistic extension of the traditional product-of-coefficients method will be used to ascertain each identified feature’s TIE and CIE. First, a marginal linear regression will model the effect of the exposure on each feature and the effect of each feature on the outcome, controlling for the exposure. The TIE will be calculated as the sum of the CIE. The CIE will be calculated as the product of the coefficients from the two-step regression process. This method was implemented in R.

### Data Generation

Data was simulated using a directed acyclic graph (DAG; Figure S2). The most exogenous variable (i.e., the variable with the fewest arrows leading to the variable), the confounding set, was simulated first, followed by the exposure, metabolites, and finally the outcome. Related dependencies and distributions were induced using the following equations:

*C* = *C*_0_, where *C*_0_ was the confounder list generated from a standard normal distribution, *N*(0,1).

*E* = *E*_0_ + 0.2*C*_*i*_, where *E*_0_ was the exposure generated from a standard normal distribution, *N*(0,1), and *C*_*i*_ was the number of confounders 1···*i*. The beta value of 0.2 was selected to provide a consistent, moderate effect size across all scenarios tested.

Metabolites could be independent or correlated. If independent, each metabolite was generated from a standard normal distribution, *N*(0,1). As a basis for correlated metabolite generation, we started with draws from a multivariate normal distribution with a mean of 0 and standard deviation generated from a positive definite uniform correlation matrix, with correlations between metabolites ranging from −0.42 to 0.45.^29^ Among the set of all metabolites generated, a subset was selected to mediate the relationship between our simulated exposure and the outcome. This was accomplished by modifying the mean of the normal distributions used to generate the metabolites from 0 to *M*_*j*_ = *M*_*j*_ + β*E*, where *M*_*j*_ is the number of mediators 1···*j* and β could be defined by the user in the data-generating process.

The outcome was then generated from a linear regression model defined as *O* = *O*_0_ + 0.8*E* + 0.01*C*_*i*_ + β*M*_*j*_, where *C*_*i*_ was the number of confounders 1···*i* and *M*_*j*_ is the number of mediators 1···*j*. If a metabolite was determined to be a mediator, β was defined as nonzero in the data-generating process; otherwise, the value of beta was set to 0.

### Simulation Scenarios

The simulation aimed to understand how high-dimensional mediation techniques behaved in an untargeted metabolomic setting, which have been applied in previous research.^2,10,19,23,24^ The simulation scenarios described below were designed with this scenario in mind. We used 1000 Monte Carlo simulations to assess the performance of each method.^30,31^

To understand the impact sample size has on the different high-dimensional mediation techniques, we simulated a small sample size of 250 individuals, a medium sample size of 500 individuals, and a large sample size of 1000 individuals. These sample sizes were chosen as they are reflective of or larger than what is typically seen in untargeted metabolomic analyses. This can demonstrate how the efficiency and accuracy of these techniques might change with an increased power as the field matures. The size of the mediating set was varied to 200, 400, and 600 to understand how a changing number of metabolites impacts the behavior of the techniques. Next, research suggests that the number of true mediators is a small percentage of the actual number of identified metabolites.^2,19,32^ To reflect this, 2, 5, and 10% of the metabolites were set as true mediators. Next, the β values of the exposure-to-mediator and mediator-to-outcome relationship were set to either 0.1 or 0.3 to reflect the relatively small beta values observed in metabolomics studies and to see how this impacts each method’s ability to identify the mediating set. Finally, all scenarios were run with independent and correlated mediating sets to understand how violation of exchangeability assumption 4 impacts HIMA, HDMA, and MITM. To conduct the simulation, we used a high-performance computing cluster composed of Dell Power Edge systems equipped with Intel Xeon processors, with a total of 32 cores and 100GB memory. Simulating correlated high-dimensional metabolomics data sets, and running 1000 replicates of each scenario was not feasible using the memory and processing capabilities of a standard laptop. Use of the cluster allowed us to complete the computation efficiently, without restricting personal computing availability. Note that the mediation analyses tools examined in this study operate comparably on standard hardware and do not inherently require HPC environments when applied to real data sets.

To improve figure clarity, only the results for the largest mediator set (*p* = 600) are presented in the main text, as it most closely approximates dimensionality common in untargeted metabolomics and provides a conservative representation of method performance. Results for *p* = 200 and *p* = 400 are included in the Supporting Information, as similar performance trends were observed across mediator set sizes.

### Analysis

This study utilized several key evaluation metrics, including the CIE, the TIE, and the sensitivity and specificity of each method, to comprehensively assess the performance of different analytical approaches and their impact on estimator accuracy.

The TIE can be defined using potential outcomes, a causal understanding underpinning this effort.^33,34^ Define *X* as the exposure, *Y* as the outcome, and *M* as the mediator. The TIE can be defined as follows:

$$E\left[YxMx-YxMx^{*}\right]=\Sigma mE\left[Y|x,m\right]\left\{P\left(m|x\right)-P\left(m|x^{*}\right)\right\}$$

The left-hand side of the equation, *E*[*YxMx* – YxMx*], represents the difference in the potential outcomes (*Y*) when the exposure (*x*) is held constant and when the mediators (*M*) are held at varying exposure levels (*x* or x*). On the right-hand side of the equation, the expression Σ*mE*[*Y* | *x*, *m*] is the expected outcome when both the exposure (*x*) and mediator (*m*) are set to specific levels summed over all mediator values. The summation is weighted by the probability, {*P*(*m* | *x*) – *P*(*m* | x*)}, which represents the difference in the probability of the mediator (*M*) taking on a specific value (*m*) under the two exposure scenarios (*x* or x*).

As the TIE captures all information explained by the set of metabolites between the exposure and outcome, the correlation structure does not impact the causal interpretation of this estimand. However, the CIE can be interpreted only in scenarios where you can assume independence. If there are dependencies in the data, isolating the effect of a single component (e.g., for *M*_1_) would require the following causal comparison:

$$E\left[YxM_{1}xM_{2}x\cdot \cdot \cdot M_{j}x-YxM_{1}x^{*}M_{2}x\cdot \cdot \cdot M_{j}x\right]$$

This contrast is not possible if *M*_1_ is dependent on any other mediator, as information from the exposure indicates that the exposure status is exposed (x*), but information from all other mediators (*M*_2_ *x*···*M*_*j*_ *x*) indicates that the exposure status is unexposed (*x*). This impossible paradox makes the causal contrast uninterpretable, due to the “recanting witness” problem.^35,36^ To deal with this issue, we evaluated the CIE only in scenarios where independence between mediators is assumed.

To evaluate the performance of the mediation methods HDMA, HIMA, and MITM, we assessed their sensitivity and specificity in identifying the true mediators from the overall set. Sensitivity was defined as the number of correctly identified mediators divided by the total number of true mediators for each simulation. Specificity was defined as the number of correctly rejected nonmediators divided by the total number of nonmediators for each simulation. These metrics allowed us to quantitatively compare the effectiveness of each method in accurately identifying true mediators and correctly rejecting nonmediators within simulated metabolomics data. The analysis was conducted in R (version 4.4.1).

To evaluate the performance of each mediation method, we simulated 36 scenarios for each level of mediator set size (*p* = 200, 400, 600). These scenarios represent all combinations of the following parameters: correlation structure of the mediators (correlated or independent), sample size (*n* = 200, 500, 1000), the proportion of true mediators (2, 5, or 10% of *p*), and the effect size of the exposure-to-mediator and mediator-to-outcome relationships (β = 0.1 or 0.3). Scenarios 1–18 represent the correlated mediator settings, while scenarios 19–36 correspond to the independent mediator settings. Additionally, scenarios 1–9 and 19–27 used a smaller mediator beta value (β = 0.1), while the remaining scenarios used a larger beta value (β = 0.3). Bias was calculated as the difference between the mean estimated effect across simulation replicates and the corresponding true effect specified in the data-generating process. This structure allowed us to systematically assess how each method performs under varying data dimensionality and effect size conditions.

## Results

We present simulation results on how the methods performed at estimating the CIE, the TIE, and sensitivity and specificity. Within each section, results are presented first for scenarios with an independent mediator structure, followed by those with a correlated mediator structure (if applicable).

### CIE

The CIE was evaluated only for the independent metabolite scenarios. For all simulation scenarios tested in which metabolites were assumed to be independent, results did not drastically differ across HDMA, HIMA, and MITM (Figure 1 and Figure S3 and Table S1). Generally, HDMA and HIMA were both unbiased for the CIE in the independent metabolite setting. In contrast, MITM appeared to systematically overestimate the CIE in settings with smaller betas (β = 0.1; Figure 1 and Figure S3, scenario 19–27) and systematically underestimate the CIE in settings with larger mediator betas (β = 0.3, Figure 1 and Figure S3, scenario 20–36). Mediator betas refer to the fixed values used in the data-generating process to represent the strength of the exposure-to-mediator and mediator-to-outcome relationships. The magnitude of the bias in estimating the CIE for the MITM approach ranged from −0.001 to 0.058 for settings with smaller betas and from −0.075 to −0.013 for settings with larger betas (Table S1).

**Figure 1:**
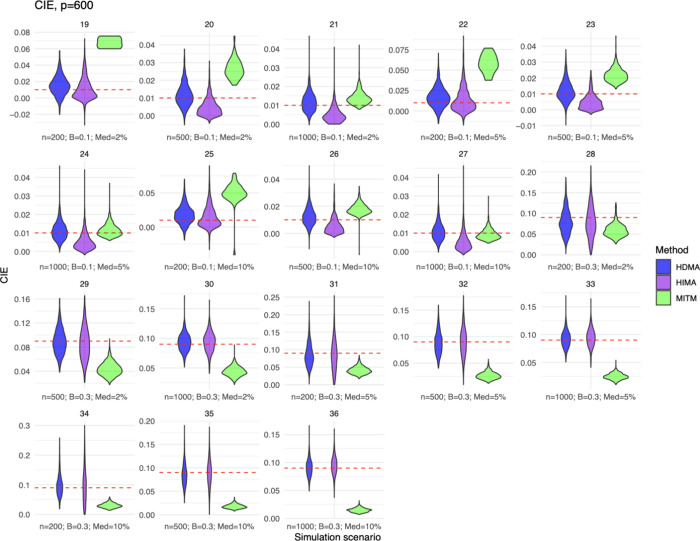
Violin density plots of the component indirect effect (CIE) estimates among independent scenarios (numbers 19–36) where the number of mediating metabolites (*p*) equals *p* = 600. Plots for *p* = 200 and *p* = 400 are in Figure S3 with similar trends and patterns highlighted here. CIE estimates for HDMA are represented by blue, HIMA purple, and MITM green. The true CIE for each simulation scenario is shown using the red dashed line. Each simulation scenario has a unique sample size (*n*), mediator beta value (*B*), and percent of true mediators (Med) combination.

### TIE

The total indirect effect was examined in scenarios where the metabolites were independent and correlated (Figure 2 and Figure S4 and Table S2). In the independent scenarios, the TIE was generally underestimated (Figure 2, all scenarios except Figure S4, scenario 19 when *p* = 200) using the MITM approach. The magnitude of bias ranged from −5.233 to 0.019 for the MITM approach (Table S2). For HIMA, the TIE was underestimated in the lower mediator beta settings (β = 0.1; Figure 2 and Figure S4), but generally improved at β = 0.3. The magnitude of the bias in estimating the TIE for the HIMA approach ranged from −0.547 to −0.027 for settings with smaller betas and −3.43 to 0.171 for settings with larger betas (Table S2). HDMA could estimate the TIE in most independent scenarios, except for scenarios 25 and 34 at *p* = 400 and 600, both of which were high-dimensional settings (Figure 2 and Figure S4). The magnitude of bias ranged from −3.109 to 0.046 for the HDMA approach (Table S2). Across all of the independent scenarios, methods performed better when the number of simulated mediators (*p*) was smaller than the sample size (*n*), i.e., in low-dimensional simulation settings.

**Figure 2:**
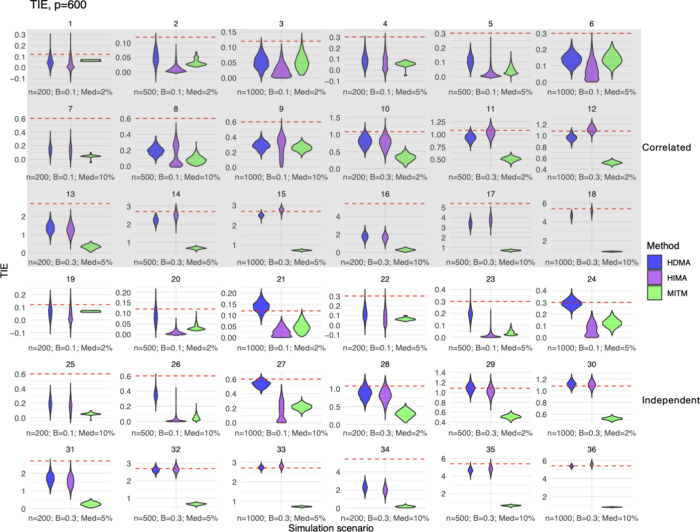
Violin density plots of the total indirect effect (TIE) estimates where the number of mediating metabolites (*p*) equals *p* = 600. Plots for *p* = 200 and *p* = 400 are in Figure S4 with similar trends and patterns highlighted here. TIE estimates for HDMA are represented by blue, HIMA purple, and MITM green. The true TIE for each simulation scenario is shown using the red dashed line. Each simulation scenario has a unique sample size (*n*), mediator beta value (*B*), and percent of true mediators (Med) combination. Correlated scenarios have a gray background (scenarios 1–18), and independent scenarios have a white background (scenarios 19–36).

There were similar results in correlated scenarios (Figure 2 and Figure S4), with the TIE underestimated for MITM and the performance worsened as the number of metabolites increased. The magnitude of bias ranged from −5.113 to −0.005 for the MITM approach (Table S2). For HDMA and HIMA, the sample size (*n*) had less of an impact on the underestimation of the TIE in the lower mediator beta settings (β = 0.1) compared to the independent scenarios. When β = 0.3, HIMA performed the most consistently, although smaller sample sizes and high-dimensional scenarios (i.e., when *p* > *n*) caused this method to underestimate the TIE. In this setting, HDMA appeared to be more affected by the correlated data structure compared with HIMA, resulting in less accurate estimates. The magnitude of the bias in estimating the TIE for the HIMA approach ranged from −3.751 to 0.07 in correlated settings, and for HDMA, it ranged from −3.632 to −0.014 in correlated settings (Table S2).

### Sensitivity and Specificity

Sensitivity had a wide range, depending on the scenario being tested (<25 to 100% of true mediators captured). Sensitivity improved for all methods as sample size increased, with larger mediator beta values, and in low-dimensional settings (Figure 3 and Figure S5 and Table S3). In contrast, specificity was consistently high across all scenarios for all methods (>90%; Figure 4 and Figure S6 and Table S3). MITM tended to have lower sensitivity compared to HIMA and HDMA, especially in lower mediator beta settings (<60%), but generally the highest specificity (>97%). HIMA and HDMA tended to be comparable for sensitivity, but HMA consistently had higher specificity compared to HDMA, although both methods were above 90%.

**Figure 3:**
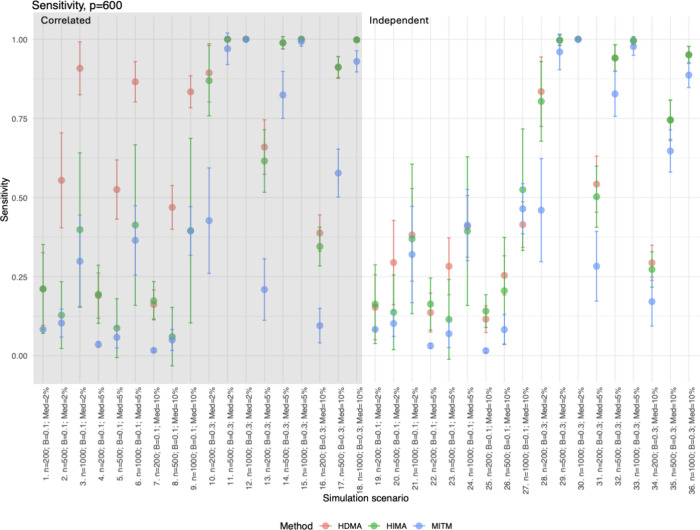
Sensitivity for *p* = 600. Plots for *p* = 200 and *p* = 400 are in Figure S5 with similar trends and patterns highlighted here. The points represent the mean, and the whiskers are the standard deviation for each simulation scenario (1000 repeats per scenario). Correlated scenarios have a gray background (scenarios 1–18), and independent scenarios have a white background (scenarios 19–36).

**Figure 4:**
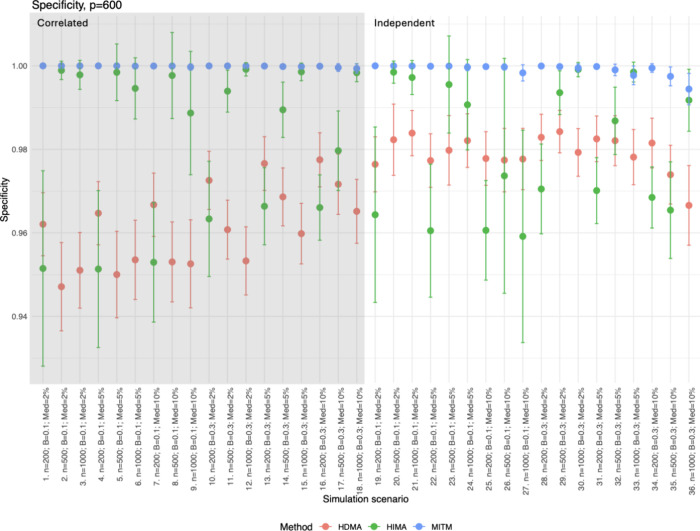
Specificity for *p* = 600. Plots for *p* = 200 and *p* = 400 are in Figure S6 with similar trends and patterns highlighted here. The points represent the mean, and the whiskers are the standard deviation for each simulation scenario (1,000 repeats per scenario). Correlated scenarios have a gray background (scenarios 1–18), and independent scenarios have a white background (scenarios 19–36).

## Discussion

Given the rapidly growing application of metabolomics in epidemiology and environmental health research, it is critical to identify efficient and accurate mediation methods to investigate biological underpinnings mediating exposure–outcome relationships. In our study, we compared and evaluated HIMA,^6^ HDMA,^7^ and MITM^8^ approaches in simulated metabolomics data to systematically examine their performance in different scenarios, especially when the mediating set is correlated to better model the complex biological interactions inherent in metabolomics data. In the correlated settings, methods cannot estimate the CIE as there is no clear causal contrast, and methods tend to underestimate the TIE. In lower mediator beta settings (β = 0.1) with a small sample size and/or a high number of potential mediating metabolites, sensitivity dropped to about 50% or less. However, while many of the true mediating metabolites were not identified (as reflected by the low sensitivity), all methods robustly identified true mediating metabolites, as the specificity was >90% for all methods.

Based on these results, researchers interested in pursuing high-dimensional mediation analyses with HIMA,^6^ HDMA,^7^ and MITM^8^ approaches in metabolomics data may want to consider incorporating multiple approaches into their workflow. Although HIMA demonstrated the strongest overall performance and may be the preferred method, it is more complex to implement and interpret compared to MITM. Therefore, using methods such as MITM alongside HIMA could be advantageous, as it allows researchers to identify and cross-validate key metabolites, highlighting those found by both methods as potentially more reliable biological signals.

Applications of these high-throughput mediation methods can be found in recent studies. While HIMA and HDMA have mainly been applied in epigenetics research, they could also be applied by researchers to metabolomics work, as the MITM approach has.^7^ ^,^ ^12,37^ For instance, one recent study applied HIMA^6^ to identify potential intermediate metabolites that could provide biological context to the association between smoking and esophageal squamous cell carcinoma, highlighting glutamine, histidine, and cholic acid as potential biological mediators.^11^ Another study used the MITM^8^ approach and found that higher serum concentrations of two per- and polyfluorinated substance (PFAS) congeners (perfluorooctanoic acid [PFOA] and perfluorononanoic acid [PFNA]) were associated with fetal growth and that this statistical association might be partially explained by perturbations of metabolites, including glycine, taurine, uric acid, ferulic acid, 2-hexyl-3-phenyl-2-propenal, unsaturated fatty acid C18:1, androgenic hormone conjugate, parent bile acid, and bile acid-glycine conjugate that were associated with both the exposure and outcome.^10^ Another study applying MITM identified tryptophan metabolism, specifically tryptophan, tyrosine, thyroxine, and serine, as biological intermediates between maternal phthalate exposure and infant neurobehavioral outcomes.^37^ While these studies demonstrate feasibility, their reliance on a single mediation framework limits the robustness of the findings. In this simulation study, we find that direct interpretation of the component indirect effect (CIE) or total indirect effect (TIE) becomes increasingly unreliable under less favorable conditions, particularly when the sample size is small and mediator effect sizes (β) are weak, as is common in real-world metabolomics analyses. These findings underscore the limitations of relying on a single mediation approach, as was done in prior studies. For example, the MITM examples had small sample sizes and used unadjusted *p*-values, raising the possibility of false positives. By contrast, incorporating a high-dimensional mediation method alongside MITM could provide greater confidence in identified mediators, balancing the flexibility of MITM with the stricter variable selection procedures of HIMA or HDMA. At the same time, the study that used HIMA alone may have missed meaningful metabolites during the dimension-reduction step, especially if true mediators exerted weaker effects. Together, these limitations highlight why a single approach may yield incomplete results and why complementary strategies are recommended.

An example of parallel approaches in metabolomics applied both HDMA and MITM to identify intermediate metabolites linking particulate matter (PM_2.5_) exposure to early birth.^38^ In this study, perturbations in protein digestion and absorption, as well as aromatic amino acids (phenylalanine, tyrosine, and tryptophan), were identified by both methods. Because these findings converged across two distinct analytic frameworks, they can be considered more robust than if either method had been used alone, reducing concerns about false positives from MITM or missed signals from HDMA. This illustrates the strength of parallel approaches in providing greater confidence in metabolomic mediation findings.

Simulations are simplifications of what might be observed in a real-world data analysis, and in this study, several simplifications were made that might not be representative of real metabolomic data. First, the mediating sets explored here had *p* = 200, 400, and 600 metabolites, whereas a real-world metabolomics mediation analysis might see a mediating set closer to 10,000. Simulating larger, correlated data sets exceeded the memory capacity and runtime limits of our computing cluster (comprised of Dell Power Edge systems equipped with Intel Xeon processors, with a total of 32 cores and 100GB memory), which restricted us from scaling the number of mediators to levels typically observed in real-world metabolomics data. However, the trends observed in this study regarding high dimensionality and sample size are not expected to improve as the value of *p* increases; in fact, our results are likely conservative, as encountering performance limitations with 600 mediators strongly suggests that the challenges would only worsen in real-world metabolomics data sets with 10,000 or more features at comparable sample sizes. Next, we applied a correlation matrix to model relationships between metabolites rather than defining each causal connection explicitly, as mapping out every causal relationship would have been time-consuming and computationally infeasible for this study. The correlation matrix was a practical approximation, allowing us to simulate high-dimensional data while maintaining a realistic representation of the metabolite interactions. However, it is important to acknowledge that this simplification does not fully capture the intricate causal dependencies inherent in biological systems. Future studies could explore more sophisticated models that better reflect these causal relationships, although such approaches will require significant computational resources and time. We also assumed no unmeasured or uncontrolled confounding in the simulation and set the mediator beta values to fixed values rather than allowing them to vary, as they would in reality. This controlled approach enabled us to specifically examine the effects of low versus high mediator beta values on the performance of the mediation methods in an unbiased setting. Finally, we limited our study to two commonly used high-dimensional mediation methods, HIMA and HDMA, acknowledging that this is a rapidly evolving field with many other emerging approaches that warrant future investigation. Additionally, our simulations did not explicitly account for the technical complexities inherent to untargeted metabolomics data, such as redundant ion species or instrumental drift. Redundant ion species, which often arise from adducts, isotopes, or in-source fragments, can be highly correlated with one another. These redundancies increase the proportion of correlated features in the data set, potentially amplifying challenges for high-dimensional mediation methods. Similarly, instrumental drift and other sources of technical variability can introduce structured noise that influences statistical inference. While our correlation matrix approach approximated intermetabolite relationships, it did not incorporate these technical artifacts. Future simulation studies could explicitly model redundant ions or drift effects to evaluate their impact on the mediation performance. Such efforts would provide actionable insights for improving data preprocessing and feature selection strategies in metabolomics, ultimately ensuring that downstream statistical analyses are more robust to technical noise. Despite these considerations, this work can be applied to studies across the field and gives researchers a clearer understanding of the analytical tools at their disposal and the drawbacks and benefits of these tools in different study settings.

At a broad level, the analytical tools available to researchers planning to conduct high-dimensional mediation in metabolomics data are not ideal, highlighting a pressing need to develop new methods that better fit the considerations of this kind of data. Methods that are developed should be robust to correlations or causal relationships in the mediating set. Additionally, there is potential for new approaches that focus on the metabolomic pathway level rather than individual metabolites, which would better capture the broader biological context and interactions. Lastly, improving computational efficiency and scalability for high-dimensional data remains a key challenge, particularly when sample sizes are small relative to the number of mediators.

The comparison of the HIMA,^6^ HDMA,^7^ and MITM^8^ approaches provides researchers with a foundation for applying current methodologies appropriately and for improving mediation analyses in metabolomics. Although current methods can provide researchers with an understanding of biological signals that could be further researched, further work and development are needed in this area to refine the methods for causal applications.

## Supplementary Material



## Data Availability

All code related to this project can be found on the following GitHub repository: https://github.com/ssb214/mediation_sim_aim2/tree/main

## Notes

Overview of the different effects (total indirect effect, direct effect, and component indirect effect) that were examined in this research (Figure S1); directed acyclic graph (DAG) that was used to build the simulation (Figure S2); component indirect effects (CIE), total indirect effects (TIE), sensitivity, and specificity plots for p = 200 and p = 400 (Figure S3, Figure S4, Figure S5, and Figure S6, respectively); CIE values (as shown in Figure 1 and Figure S3) (Table S1), TIE values (as shown in Figure 2 and Figure S4) (Table S2), and sensitivity and specificity values (as shown in Figures 3,4, S5 and S6) (Table S3) (PDF)
