# Supplementary material for: Evaluating Methods for High-Dimensional Mediation in Metabolomics Data
Source: Environ Sci Technol. 2026 Jan 7;60(2):1913–23. doi: 10.1021/acs.est.5c09706 (PMC12825160; doi:10.1021/acs.est.5c09706)
Supplement: Supplementary file 1 [file es5c09706_si_001.pdf]

Supplementary Information

Title: Evaluating methods for high-dimensional mediation in metabolomics data

Authors:

Susan S. Hoffman[1], Donghai Liang*[2,1], Anne Dunlop[3], Todd Everson[2], Audrey J. Gaskins[1], Dean P. Jones[4], Anke Hüls[1,2], Michele Marcus[1,2], Ashley I Naimi*[1]

[1]Department of Epidemiology, Emory University, Atlanta, GA, 30322, USA
[2]Gangarosa Department of Environmental Health, Emory University, Atlanta, GA, 30322, USA
[3]Department of Gynecology and Obstetrics, School of Medicine, Emory University, Atlanta, GA, 30322, USA
[4]School of Medicine, Emory University, Atlanta, GA, 30322, USA

**Corresponding authors:*

Donghai Liang
Gangarosa Department of Environmental Health; Department of Epidemiology
Emory University Rollins School of Public Health
1518 Clifton Rd, Rm 2037
Atlanta, GA 30322, USA
donghai.liang@emory.edu

Ashley Naimi
Department of Epidemiology
Emory University Rollins School of Public Health
1518 Clifton Rd, Rm XXXX
Atlanta, GA 30322, USA
Ashley.naimi@emory.edu

This file contains two figures (Figure S1 and S2) and three tables (Table S1, S2, and S3). Figure S1 is an overview of the different effects (total indirect effect, direct effect, and component indirect effect) that were examined in this research. Figure S2 is the directed acyclic graph (DAG) that was used to build the simulation. The tables provide a summary of the information visualized in the main figures. Table S1 summarizes the component indirect effects (as shown in Figure 1), Table S2 the total indirect effects (as shown in Figure 2), and Table S3 the sensitivity and specificity (as shown in Figures 3 and 4).

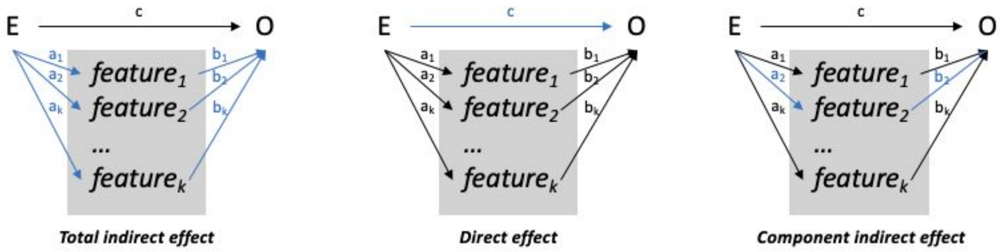

Figure S1. The total indirect effect (TIE) is the effect of the exposure to the outcome through the mediating metabolomic features (i.e., routes a_1b_1, a_2b_2,…, a_kb_k). The direct effect represents the effect of the exposure to the outcome through all pathways not contained in the mediating set (i.e., route c). The component indirect effect (CIE) is the effect of the exposure on the outcome through some specific metabolic feature (e.g., route a_1b_1, etc.,).

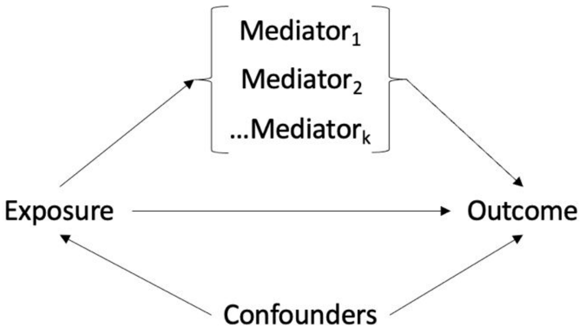

Figure S2. Directed acyclic draft (DAG) of the general data generating structure for the simulation study. The confounding set is the most exogenous variable and is generated first, then the exposure, mediating set, and finally, the outcome. The arrows represent the causal dependencies between each variable induced in the data generation. The mediating set captured in the brackets could be either independent of each other or correlated (negatively and positively).

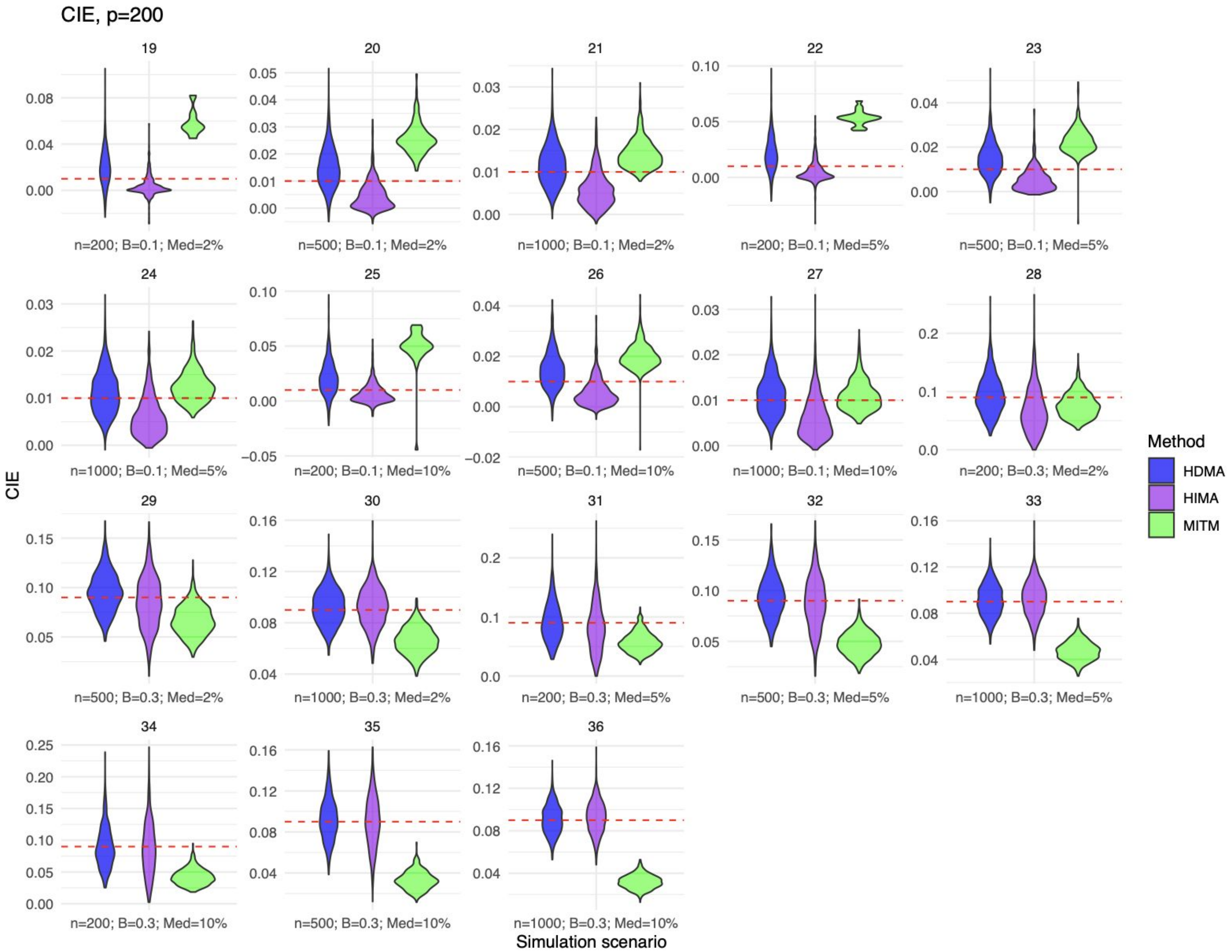

CIE, p=200
19
20
21
22
23
n=200; B=0.1; Med=2%
n=500; B=0.1; Med=2%
n=1000; B=0.1; Med=2%
n=200; B=0.1; Med=5%
n=500; B=0.1; Med=5%
24
25
26
27
28
n=1000; B=0.1; Med=5%
n=200; B=0.1; Med=10%
n=500; B=0.1; Med=10%
n=1000; B=0.1; Med=10%
n=200; B=0.3; Med=2%
29
30
31
32
33
n=500; B=0.3; Med=2%
n=1000; B=0.3; Med=2%
n=200; B=0.3; Med=5%
n=500; B=0.3; Med=5%
n=1000; B=0.3; Med=5%
34
35
36
n=200; B=0.3; Med=10%
n=500; B=0.3; Med=10%
n=1000; B=0.3; Med=10%
CIE
Simulation scenario
Method
HDMA
HIMA
MITM

(A)

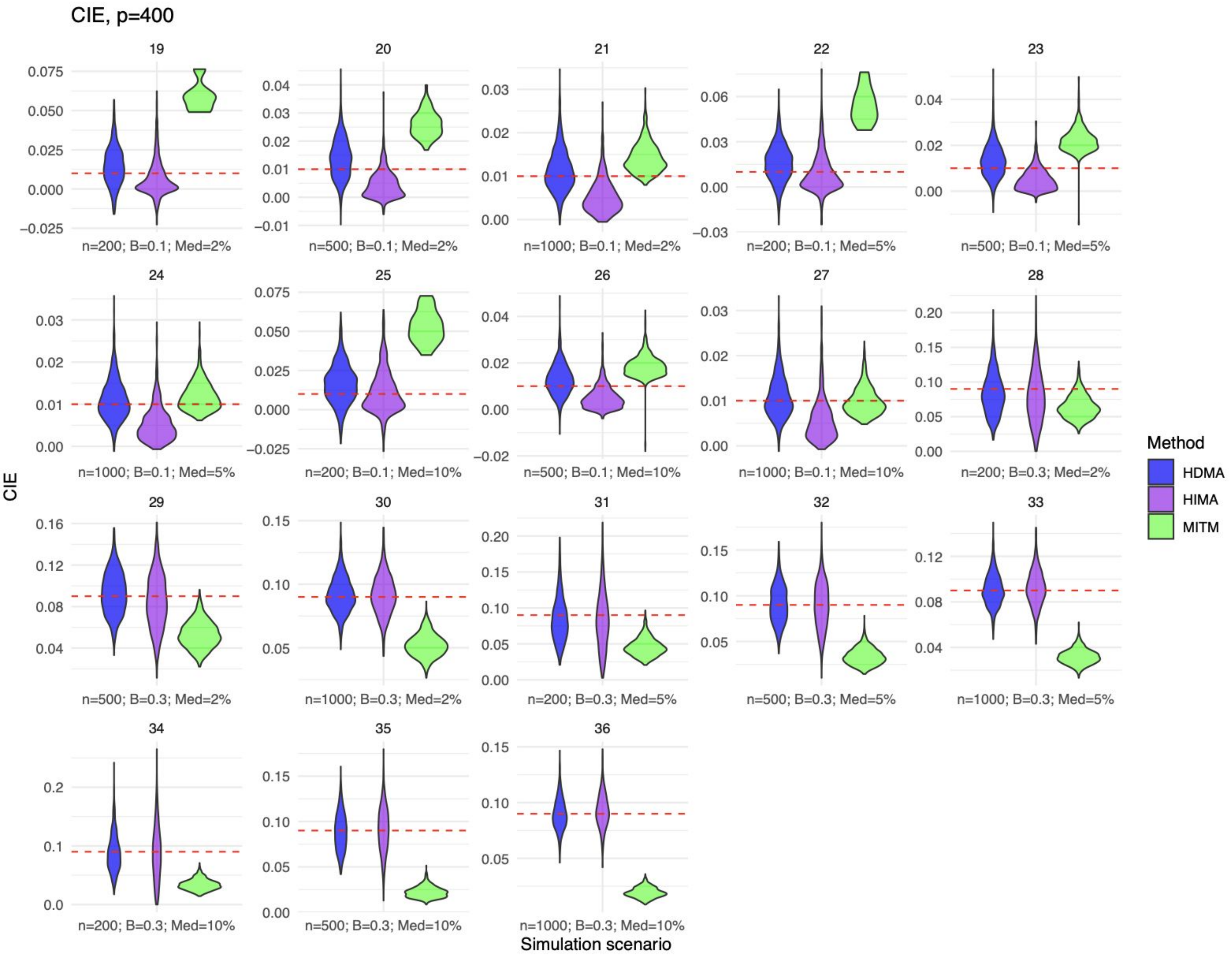
CIE, p=400
19
20
21
22
23
24
25
26
27
28
29
30
31
32
33
34
35
36
n=200; B=0.1; Med=2%
n=500; B=0.1; Med=2%
n=1000; B=0.1; Med=2%
n=200; B=0.1; Med=5%
n=500; B=0.1; Med=5%
n=1000; B=0.1; Med=5%
n=200; B=0.1; Med=10%
n=500; B=0.1; Med=10%
n=1000; B=0.1; Med=10%
n=200; B=0.3; Med=2%
n=500; B=0.3; Med=2%
n=1000; B=0.3; Med=2%
n=200; B=0.3; Med=5%
n=500; B=0.3; Med=5%
n=1000; B=0.3; Med=5%
n=200; B=0.3; Med=10%
n=500; B=0.3; Med=10%
n=1000; B=0.3; Med=10%
CIE
Simulation scenario
Method
HDMA
HIMA
MITM

(B)

Figure S3. Violin density plots of the component indirect effect (CIE) estimates among independent scenarios (numbers 19-36) where the number of mediating metabolites (p) equals (A) p = 200 and (B) p = 400. The CIE for p = 600 is reflected in the main text in Figure 1 with similar trends and patterns highlighted here. CIE estimates for HDMA are represented by blue, HIMA purple, and MITM green. The true CIE for each simulation scenario is shown using the red dashed line. Each simulation scenario has a unique sample size (n), mediator beta value (B), and percent of true mediators (Med) combination.

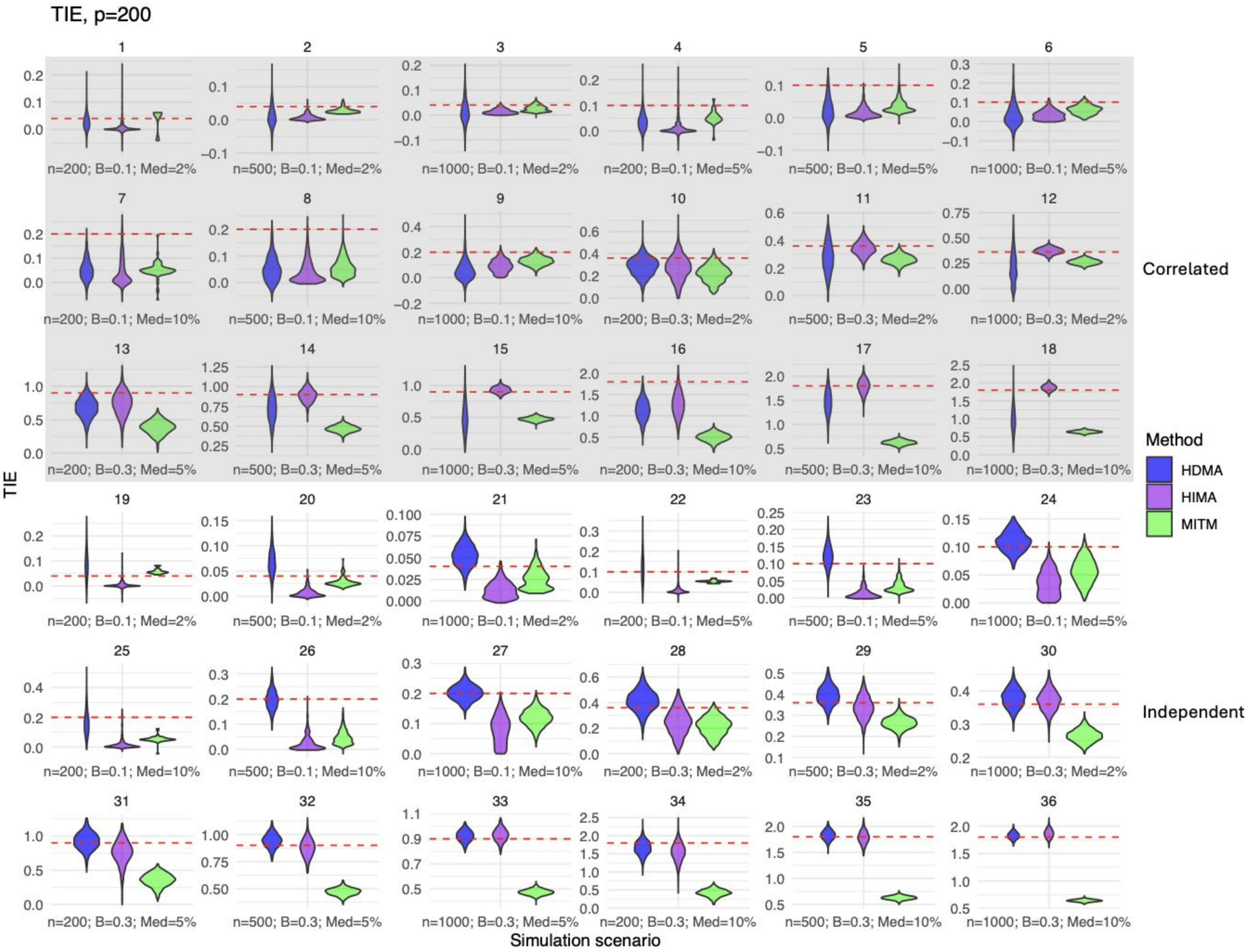

TIE, p=200
TIE
Simulation scenario
Correlated
Independent
Method
HDMA
HIMA
MITM
n=200; B=0.1; Med=2%
n=500; B=0.1; Med=2%
n=1000; B=0.1; Med=2%
n=200; B=0.1; Med=5%
n=500; B=0.1; Med=5%
n=1000; B=0.1; Med=5%
n=200; B=0.1; Med=10%
n=500; B=0.1; Med=10%
n=1000; B=0.1; Med=10%
n=200; B=0.3; Med=2%
n=500; B=0.3; Med=2%
n=1000; B=0.3; Med=2%
n=200; B=0.3; Med=5%
n=500; B=0.3; Med=5%
n=1000; B=0.3; Med=5%
n=200; B=0.3; Med=10%
n=500; B=0.3; Med=10%
n=1000; B=0.3; Med=10%

(A)

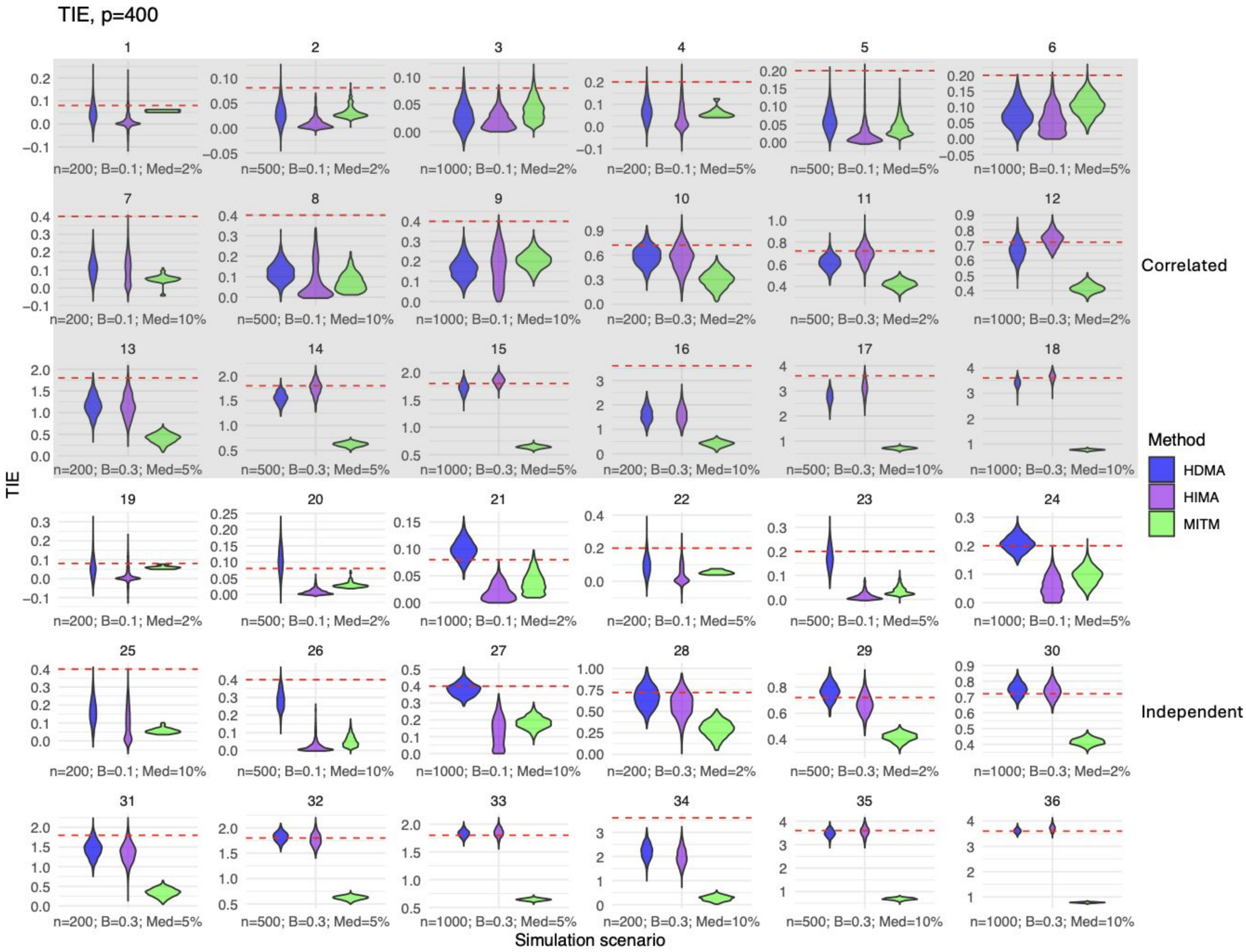
TIE, p=400
1
n=200; B=0.1; Med=2%
2
n=500; B=0.1; Med=2%
3
n=1000; B=0.1; Med=2%
4
n=200; B=0.1; Med=5%
5
n=500; B=0.1; Med=5%
6
n=1000; B=0.1; Med=5%
7
n=200; B=0.1; Med=10%
8
n=500; B=0.1; Med=10%
9
n=1000; B=0.1; Med=10%
10
n=200; B=0.3; Med=2%
11
n=500; B=0.3; Med=2%
12
n=1000; B=0.3; Med=2%
Correlated
13
n=200; B=0.3; Med=5%
14
n=500; B=0.3; Med=5%
15
n=1000; B=0.3; Med=5%
16
n=200; B=0.3; Med=10%
17
n=500; B=0.3; Med=10%
18
n=1000; B=0.3; Med=10%
TIE
Method
HDMA
HIMA
MITM
19
n=200; B=0.1; Med=2%
20
n=500; B=0.1; Med=2%
21
n=1000; B=0.1; Med=2%
22
n=200; B=0.1; Med=5%
23
n=500; B=0.1; Med=5%
24
n=1000; B=0.1; Med=5%
25
n=200; B=0.1; Med=10%
26
n=500; B=0.1; Med=10%
27
n=1000; B=0.1; Med=10%
28
n=200; B=0.3; Med=2%
29
n=500; B=0.3; Med=2%
30
n=1000; B=0.3; Med=2%
Independent
31
n=200; B=0.3; Med=5%
32
n=500; B=0.3; Med=5%
33
n=1000; B=0.3; Med=5%
34
n=200; B=0.3; Med=10%
35
n=500; B=0.3; Med=10%
36
n=1000; B=0.3; Med=10%
Simulation scenario

(B)

Figure S4. Violin density plots of the total indirect effect (TIE) estimates where the number of mediating metabolites (p) equals (A) p = 200 and (B) p = 400. TIE results for p = 600 are shown in Figure 2, with similar patterns and trends. TIE estimates for HDMA are represented by blue, HIMA purple, and MITM green. The true TIE for each simulation scenario is shown using the red dashed line. Each simulation scenario has a unique sample size (n), mediator beta value (B), and percent of true mediators (Med) combination. Correlated scenarios have a grey background (scenarios 1-18), and independent scenarios have a white background (scenarios 19-36).

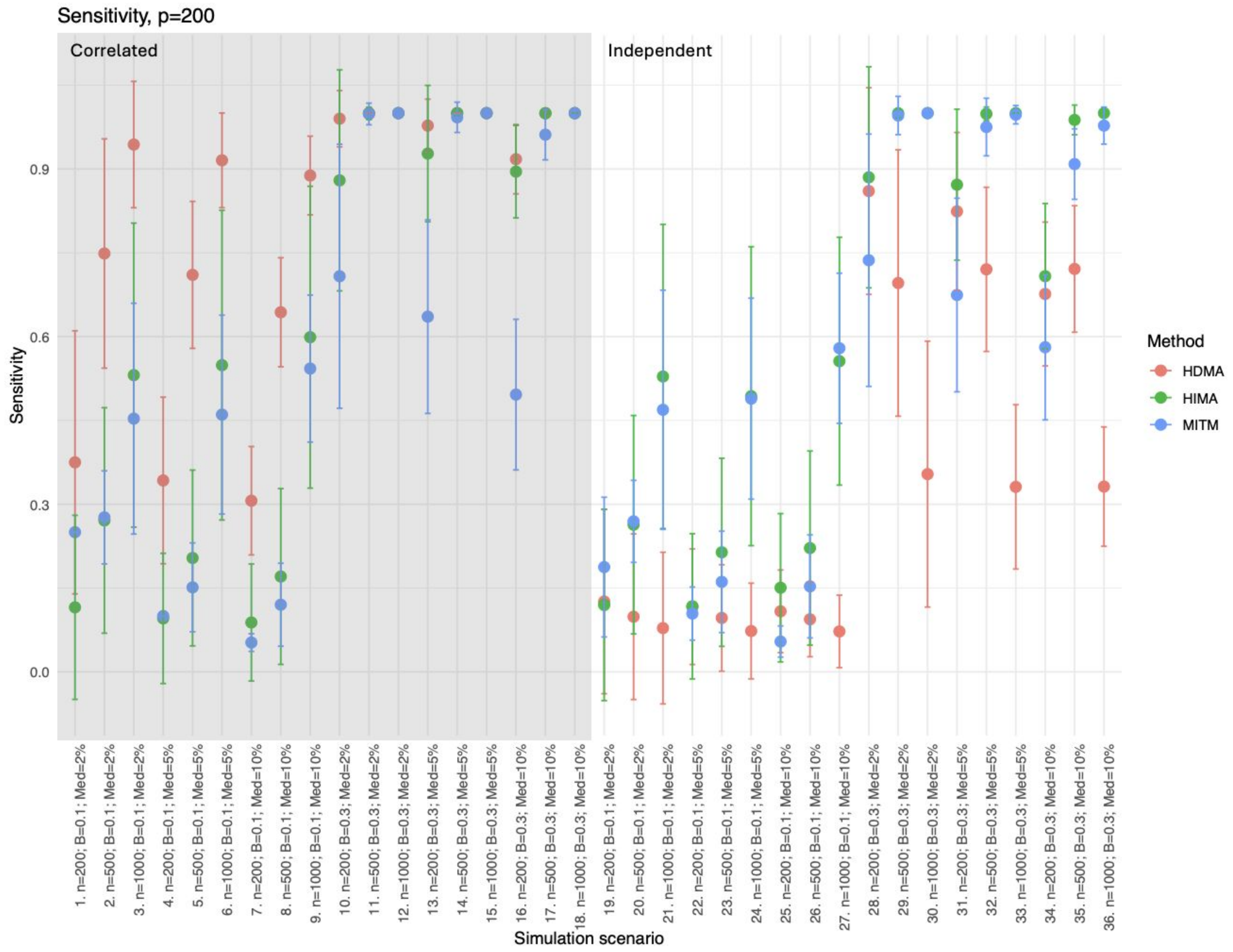

Sensitivity, p=200
Correlated
Independent
Sensitivity
0.0
0.3
0.6
0.9
Method
HDMA
HIMA
MITM
1. n=200; B=0.1; Med=2%
2. n=500; B=0.1; Med=2%
3. n=1000; B=0.1; Med=2%
4. n=200; B=0.1; Med=5%
5. n=500; B=0.1; Med=5%
6. n=1000; B=0.1; Med=5%
7. n=200; B=0.1; Med=10%
8. n=500; B=0.1; Med=10%
9. n=1000; B=0.1; Med=10%
10. n=200; B=0.3; Med=2%
11. n=500; B=0.3; Med=2%
12. n=1000; B=0.3; Med=2%
13. n=200; B=0.3; Med=5%
14. n=500; B=0.3; Med=5%
15. n=1000; B=0.3; Med=5%
16. n=200; B=0.3; Med=10%
17. n=500; B=0.3; Med=10%
18. n=1000; B=0.3; Med=10%
19. n=200; B=0.1; Med=2%
20. n=500; B=0.1; Med=2%
21. n=1000; B=0.1; Med=2%
22. n=200; B=0.1; Med=5%
23. n=500; B=0.1; Med=5%
24. n=1000; B=0.1; Med=5%
25. n=200; B=0.1; Med=10%
26. n=500; B=0.1; Med=10%
27. n=1000; B=0.1; Med=10%
28. n=200; B=0.3; Med=2%
29. n=500; B=0.3; Med=2%
30. n=1000; B=0.3; Med=2%
31. n=200; B=0.3; Med=5%
32. n=500; B=0.3; Med=5%
33. n=1000; B=0.3; Med=5%
34. n=200; B=0.3; Med=10%
35. n=500; B=0.3; Med=10%
36. n=1000; B=0.3; Med=10%
Simulation scenario

(A)

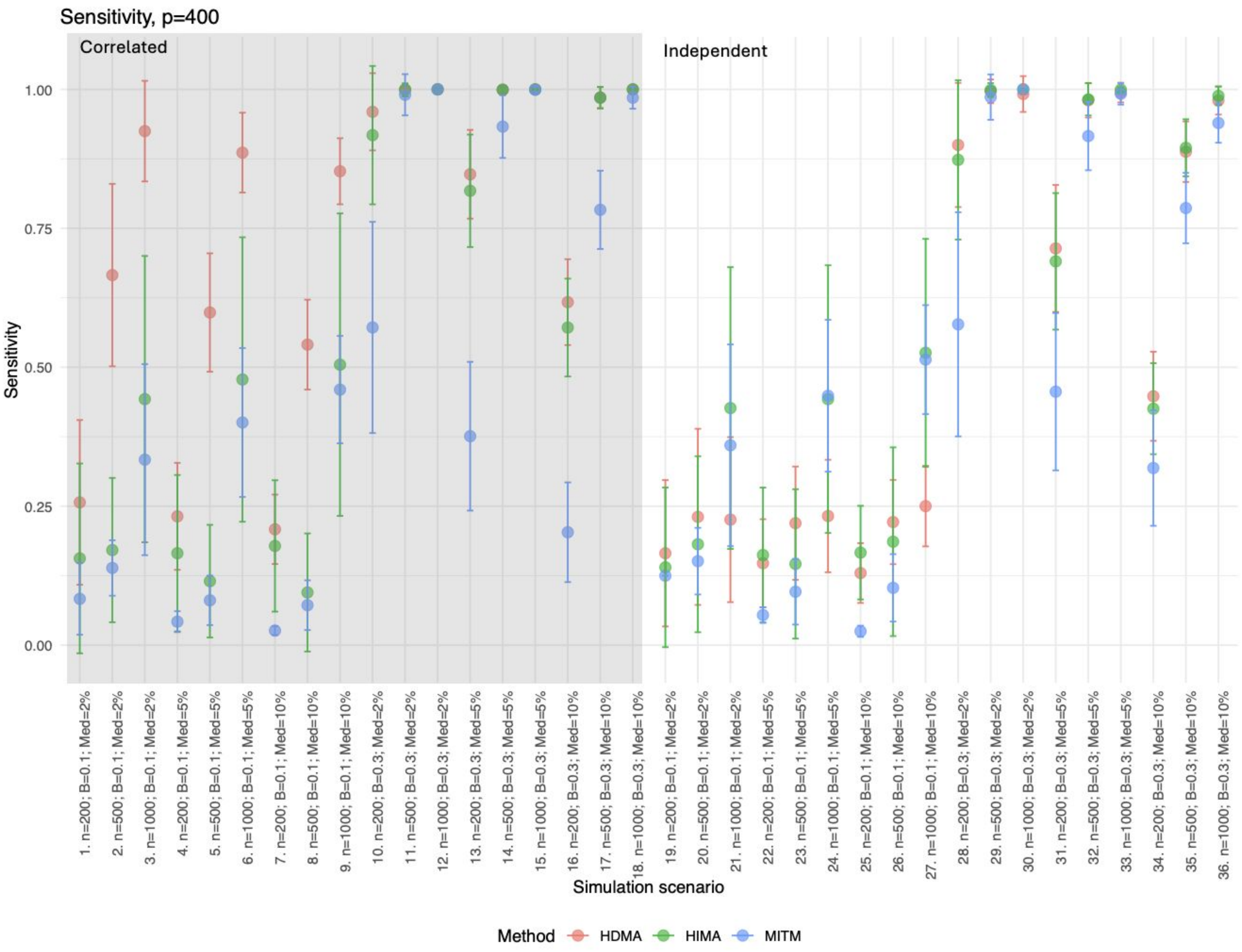
Sensitivity, p=400
Correlated
Independent
1.00
0.75
0.50
0.25
0.00
Sensitivity
1. n=200; B=0.1; Med=2%
2. n=500; B=0.1; Med=2%
3. n=1000; B=0.1; Med=2%
4. n=200; B=0.1; Med=5%
5. n=500; B=0.1; Med=5%
6. n=1000; B=0.1; Med=5%
7. n=200; B=0.1; Med=10%
8. n=500; B=0.1; Med=10%
9. n=1000; B=0.1; Med=10%
10. n=200; B=0.3; Med=2%
11. n=500; B=0.3; Med=2%
12. n=1000; B=0.3; Med=2%
13. n=200; B=0.3; Med=5%
14. n=500; B=0.3; Med=5%
15. n=1000; B=0.3; Med=5%
16. n=200; B=0.3; Med=10%
17. n=500; B=0.3; Med=10%
18. n=1000; B=0.3; Med=10%
19. n=200; B=0.1; Med=2%
20. n=500; B=0.1; Med=2%
21. n=1000; B=0.1; Med=2%
22. n=200; B=0.1; Med=5%
23. n=500; B=0.1; Med=5%
24. n=1000; B=0.1; Med=5%
25. n=200; B=0.1; Med=10%
26. n=500; B=0.1; Med=10%
27. n=1000; B=0.1; Med=10%
28. n=200; B=0.3; Med=2%
29. n=500; B=0.3; Med=2%
30. n=1000; B=0.3; Med=2%
31. n=200; B=0.3; Med=5%
32. n=500; B=0.3; Med=5%
33. n=1000; B=0.3; Med=5%
34. n=200; B=0.3; Med=10%
35. n=500; B=0.3; Med=10%
36. n=1000; B=0.3; Med=10%
Simulation scenario
Method
HDMA
HIMA
MITM

(B)

Figure S5. Sensitivity for (A) p = 200 and (B) p = 400. Sensitivity results for p = 600 are shown in Figure 3, with similar patterns and trends. The points represent the mean, and the whiskers are the standard deviation for each simulation scenario (1,000 repeats per scenario). Correlated scenarios have a grey background (scenarios 1-18), and independent scenarios have a white background (scenarios 19-36).

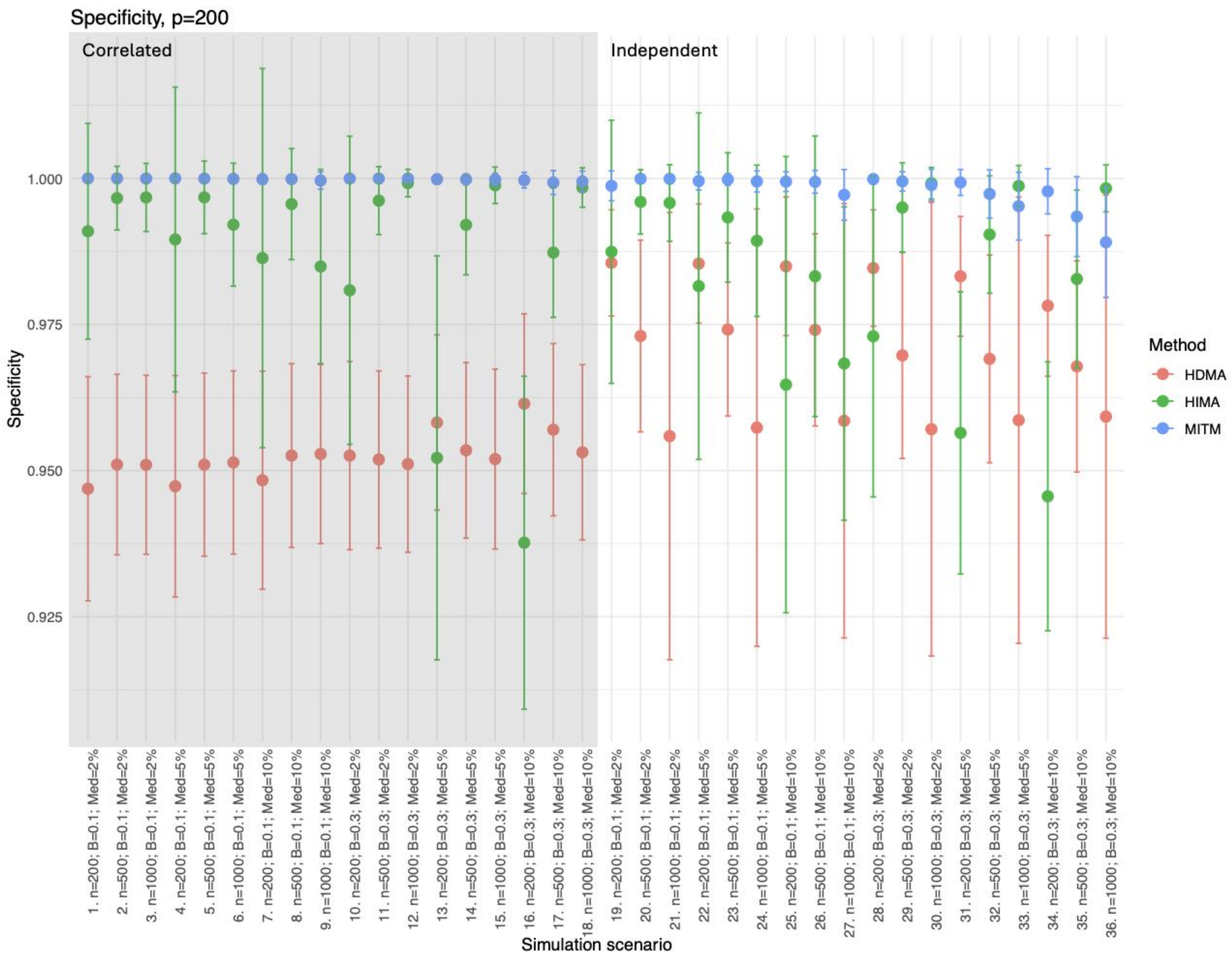

Specificity, p=200
Correlated
Independent
Specificity
1.000
0.975
0.950
0.925
Method
HDMA
HIMA
MITM
Simulation scenario
1. n=200; B=0.1; Med=2%
2. n=500; B=0.1; Med=2%
3. n=1000; B=0.1; Med=2%
4. n=200; B=0.1; Med=5%
5. n=500; B=0.1; Med=5%
6. n=1000; B=0.1; Med=5%
7. n=200; B=0.1; Med=10%
8. n=500; B=0.1; Med=10%
9. n=1000; B=0.1; Med=10%
10. n=200; B=0.3; Med=2%
11. n=500; B=0.3; Med=2%
12. n=1000; B=0.3; Med=2%
13. n=200; B=0.3; Med=5%
14. n=500; B=0.3; Med=5%
15. n=1000; B=0.3; Med=5%
16. n=200; B=0.3; Med=10%
17. n=500; B=0.3; Med=10%
18. n=1000; B=0.3; Med=10%
19. n=200; B=0.1; Med=2%
20. n=500; B=0.1; Med=2%
21. n=1000; B=0.1; Med=2%
22. n=200; B=0.1; Med=5%
23. n=500; B=0.1; Med=5%
24. n=1000; B=0.1; Med=5%
25. n=200; B=0.1; Med=10%
26. n=500; B=0.1; Med=10%
27. n=1000; B=0.1; Med=10%
28. n=200; B=0.3; Med=2%
29. n=500; B=0.3; Med=2%
30. n=1000; B=0.3; Med=2%
31. n=200; B=0.3; Med=5%
32. n=500; B=0.3; Med=5%
33. n=1000; B=0.3; Med=5%
34. n=200; B=0.3; Med=10%
35. n=500; B=0.3; Med=10%
36. n=1000; B=0.3; Med=10%

(A)

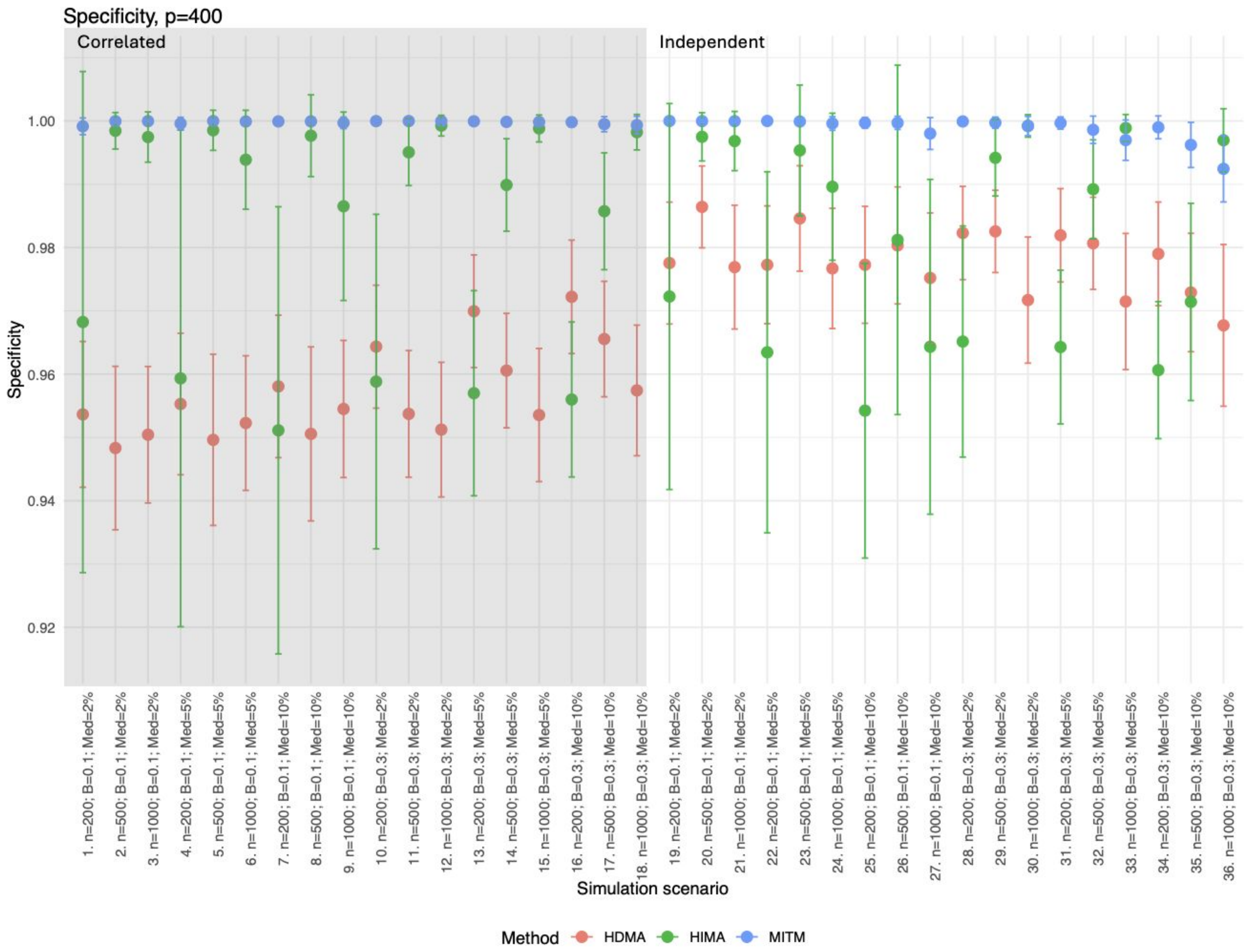

Specificity, p=400
Correlated
Independent
Specificity
1.00
0.98
0.96
0.94
0.92
1. n=200; B=0.1; Med=2%
2. n=500; B=0.1; Med=2%
3. n=1000; B=0.1; Med=2%
4. n=200; B=0.1; Med=5%
5. n=500; B=0.1; Med=5%
6. n=1000; B=0.1; Med=5%
7. n=200; B=0.1; Med=10%
8. n=500; B=0.1; Med=10%
9. n=1000; B=0.1; Med=10%
10. n=200; B=0.3; Med=2%
11. n=500; B=0.3; Med=2%
12. n=1000; B=0.3; Med=2%
13. n=200; B=0.3; Med=5%
14. n=500; B=0.3; Med=5%
15. n=1000; B=0.3; Med=5%
16. n=200; B=0.3; Med=10%
17. n=500; B=0.3; Med=10%
18. n=1000; B=0.3; Med=10%
19. n=200; B=0.1; Med=2%
20. n=500; B=0.1; Med=2%
21. n=1000; B=0.1; Med=2%
22. n=200; B=0.1; Med=5%
23. n=500; B=0.1; Med=5%
24. n=1000; B=0.1; Med=5%
25. n=200; B=0.1; Med=10%
26. n=500; B=0.1; Med=10%
27. n=1000; B=0.1; Med=10%
28. n=200; B=0.3; Med=2%
29. n=500; B=0.3; Med=2%
30. n=1000; B=0.3; Med=2%
31. n=200; B=0.3; Med=5%
32. n=500; B=0.3; Med=5%
33. n=1000; B=0.3; Med=5%
34. n=200; B=0.3; Med=10%
35. n=500; B=0.3; Med=10%
36. n=1000; B=0.3; Med=10%
Simulation scenario
Method
HDMA
HIMA
MITM

(B)

Figure S6. Specificity for (A) p = 200 and (B) p = 400. Specificity results for p = 600 are shown in Figure 3, with similar patterns and trends. The points represent the mean, and the whiskers are the standard deviation for each simulation scenario (1,000 repeats per scenario). Correlated scenarios have a grey background (scenarios 1-18), and independent scenarios have a white background (scenarios 19-36).

Table S1. Summary of component indirect effect (CIE) estimates and bias from the truth among independent scenarios. The simulation scenario column describes the unique sample size (n), mediator beta value (B), and percent of true mediators (Med). All scenarios are independent. Bias was calculated by subtracting the truth from the mean of the simulation run.

		p=200			p=400			p=600		
**Simulation Scenario**	**Method**	**Mean (SD)**	**SE**	**Bias (Truth)**	**Mean (SD)**	**SE**	**Bias (Truth)**	**Mean (SD)**	**SE**	**Bias (Truth)**
n=200; B=0.1; Med=2%	HDMA	0.02 (0.016)	0.000	0.01 (0.01)	0.015 (0.012)	0.000	0.005 (0.01)	0.015 (0.012)	0.000	0.005 (0.01)
	HIMA	0.003 (0.008)	0.000	-0.007 (0.01)	0.007 (0.011)	0.000	-0.003 (0.01)	0.011 (0.013)	0.000	0.001 (0.01)
	MITM	0.059 (0.012)	0.000	0.049 (0.01)	0.059 (0.01)	0.000	0.049 (0.01)	0.068 (0.007)	0.000	0.058 (0.01)
n=500; B=0.1; Med=2%	HDMA	0.015 (0.007)	0.000	0.005 (0.01)	0.014 (0.007)	0.000	0.004 (0.01)	0.012 (0.006)	0.000	0.002 (0.01)
	HIMA	0.005 (0.	0.000	-0.005 (0.	0.005 (0.	0.000	-0.005 (0.	0.005 (0.	0.000	-0.005 (0.

		p=200			p=400			p=600		
**Simulation Scenario**	**Method**	**Mean (SD)**	**SE**	**Bias (Truth)**	**Mean (SD)**	**SE**	**Bias (Truth)**	**Mean (SD)**	**SE**	**Bias (Truth)**
		005)		01)	005)		01)	005)		01)
	MITM	0.026 (0.006)	0.000	0.016 (0.01)	0.026 (0.005)	0.000	0.016 (0.01)	0.026 (0.005)	0.000	0.016 (0.01)
	HDMA	0.012 (0.005)	0.000	0.002 (0.01)	0.011 (0.005)	0.000	0.001 (0.01)	0.012 (0.005)	0.000	0.002 (0.01)
n=1000; B=0.1; Med=2%	HIMA	0.006 (0.004)	0.000	-0.004 (0.01)	0.005 (0.004)	0.000	-0.005 (0.01)	0.005 (0.004)	0.000	-0.005 (0.01)
	MITM	0.014 (0.003)	0.000	0.004 (0.01)	0.014 (0.003)	0.000	0.004 (0.01)	0.014 (0.003)	0.000	0.004 (0.01)
n=200; B=0.1; Med=5%	HDMA	0.021 (0.015)	0.000	0.011 (0.01)	0.016 (0.012)	0.000	0.006 (0.01)	0.017 (0.012)	0.000	0.007 (0.01)

		p=200			p=400			p=600		
**Simulation Scenario**	**Method**	**Mean (SD)**	**SE**	**Bias (Truth)**	**Mean (SD)**	**SE**	**Bias (Truth)**	**Mean (SD)**	**SE**	**Bias (Truth)**
	HIMA	0.005 (0.009)	0.000	-0.005 (0.01)	0.009 (0.012)	0.000	-0.001 (0.01)	0.014 (0.014)	0.000	0.004 (0.01)
	MITM	0.053 (0.007)	0.000	0.043 (0.01)	0.054 (0.012)	0.000	0.044 (0.01)	0.058 (0.011)	0.000	0.048 (0.01)
	HDMA	0.015 (0.007)	0.000	0.005 (0.01)	0.013 (0.007)	0.000	0.003 (0.01)	0.012 (0.006)	0.000	0.002 (0.01)
n=500; B=0.1; Med=5%	HIMA	0.005 (0.005)	0.000	-0.005 (0.01)	0.005 (0.005)	0.000	-0.005 (0.01)	0.005 (0.005)	0.000	-0.005 (0.01)
	MITM	0.023 (0.005)	0.000	0.013 (0.01)	0.022 (0.005)	0.000	0.012 (0.01)	0.022 (0.004)	0.000	0.012 (0.01)
n=1000; B=0.	HD	0.011	0.0	0.001	0.011	0.0	0.001	0.011	0.0	0.001

		p=200			p=400			p=600		
**Simulation Scenario**	**Method**	**Mean (SD)**	**SE**	**Bias (Truth)**	**Mean (SD)**	**SE**	**Bias (Truth)**	**Mean (SD)**	**SE**	**Bias (Truth)**
1; Med =5%	MA	(0.004)	00	(0.01)	(0.005)	00	(0.01)	(0.005)	00	(0.01)
	HIMA	0.006 (0.004)	0.000	-0.004 (0.01)	0.005 (0.004)	0.000	-0.005 (0.01)	0.005 (0.004)	0.000	-0.005 (0.01)
	MITM	0.013 (0.003)	0.000	0.003 (0.01)	0.012 (0.003)	0.000	0.002 (0.01)	0.012 (0.003)	0.000	0.002 (0.01)
n=200; B=0.1; Med =10%	HDMA	0.021 (0.015)	0.000	0.011 (0.01)	0.016 (0.013)	0.000	0.006 (0.01)	0.018 (0.013)	0.000	0.008 (0.01)
	HIMA	0.007 (0.009)	0.000	-0.003 (0.01)	0.011 (0.013)	0.000	0.001 (0.01)	0.015 (0.015)	0.000	0.005 (0.01)
	MITM	0.049 (0.018)	0.000	0.039 (0.01)	0.053 (0.01)	0.000	0.043 (0.01)	0.05 (0.018)	0.000	0.04 (0.01)

		p=200			p=400			p=600		
**Simulation Scenario**	**Method**	**Mean (SD)**	**SE**	**Bias (Truth)**	**Mean (SD)**	**SE**	**Bias (Truth)**	**Mean (SD)**	**SE**	**Bias (Truth)**
n=500; B=0.1; Med =10%	HDMA	0.015 (0.007)	0.000	0.005 (0.01)	0.013 (0.007)	0.000	0.003 (0.01)	0.012 (0.007)	0.000	0.002 (0.01)
	HIMA	0.006 (0.005)	0.000	-0.004 (0.01)	0.005 (0.005)	0.000	-0.005 (0.01)	0.006 (0.006)	0.000	-0.004 (0.01)
	MITM	0.02 (0.005)	0.000	0.01 (0.01)	0.019 (0.005)	0.000	0.009 (0.01)	0.018 (0.004)	0.000	0.008 (0.01)
n=1000; B=0.1; Med =10%	HDMA	0.011 (0.004)	0.000	0.001 (0.01)	0.011 (0.005)	0.000	0.001 (0.01)	0.011 (0.005)	0.000	0.001 (0.01)
	HIMA	0.006 (0.004)	0.000	-0.004 (0.01)	0.006 (0.005)	0.000	-0.004 (0.01)	0.005 (0.005)	0.000	-0.005 (0.01)
	MITM	0.011	0.0	0.001	0.01 (0.	0.0	0 (0.	0.009	0.0	-0.00

		p=200			p=400			p=600		
**Simulation Scenario**	**Method**	**Mean (SD)**	**SE**	**Bias (Truth)**	**Mean (SD)**	**SE**	**Bias (Truth)**	**Mean (SD)**	**SE**	**Bias (Truth)**
		(0.003)	00	(0.01)	003)	00	01)	(0.003)	00	1 (0.01)
n=200; B=0.3; Med=2%	HDMA	0.095 (0.033)	0.001	0.005 (0.09)	0.083 (0.029)	0.001	-0.007 (0.09)	0.08 (0.029)	0.001	-0.01 (0.09)
	HIMA	0.068 (0.037)	0.001	-0.022 (0.09)	0.078 (0.036)	0.001	-0.012 (0.09)	0.079 (0.037)	0.001	-0.011 (0.09)
	MITM	0.077 (0.021)	0.001	-0.013 (0.09)	0.064 (0.017)	0.001	-0.026 (0.09)	0.059 (0.015)	0.000	-0.031 (0.09)
n=500; B=0.3; Med=2%	HDMA	0.096 (0.02)	0.001	0.006 (0.09)	0.093 (0.02)	0.001	0.003 (0.09)	0.089 (0.02)	0.001	-0.001 (0.09)
	HIMA	0.085	0.0	-0.00	0.085	0.0	-0.00	0.085	0.0	-0.00

		p=200			p=400			p=600		
Simulation Scenario	Method	Mean (SD)	SE	Bias (Truth)	Mean (SD)	SE	Bias (Truth)	Mean (SD)	SE	Bias (Truth)
		(0.025)	01	5 (0.09)	(0.025)	01	5 (0.09)	(0.026)	01	5 (0.09)
	MITM	0.067 (0.015)	0.000	-0.023 (0.09)	0.053 (0.013)	0.000	-0.037 (0.09)	0.044 (0.011)	0.000	-0.046 (0.09)
n=1000; B=0.3; Med=2%	HDMA	0.092 (0.014)	0.000	0.002 (0.09)	0.092 (0.014)	0.000	0.002 (0.09)	0.093 (0.014)	0.000	0.003 (0.09)
	HIMA	0.092 (0.015)	0.000	0.002 (0.09)	0.092 (0.016)	0.000	0.002 (0.09)	0.093 (0.016)	0.001	0.003 (0.09)
	MITM	0.066 (0.01)	0.000	-0.024 (0.09)	0.052 (0.009)	0.000	-0.038 (0.09)	0.044 (0.008)	0.000	-0.046 (0.09)
n=200; B=0.3;	HDMA	0.092 (0.	0.001	0.002 (0.	0.084 (0.	0.001	-0.006 (0.	0.085 (0.	0.001	-0.005 (0.

		p=200			p=400			p=600		
**Simulation Scenario**	**Method**	**Mean (SD)**	**SE**	**Bias (Truth)**	**Mean (SD)**	**SE**	**Bias (Truth)**	**Mean (SD)**	**SE**	**Bias (Truth)**
Med =5%		032)		09)	03)		09)	031)		09)
	HIMA	0.08 (0.04)	0.001	-0.01 (0.09)	0.082 (0.038)	0.001	-0.008 (0.09)	0.084 (0.042)	0.001	-0.006 (0.09)
	MITM	0.057 (0.016)	0.001	-0.033 (0.09)	0.046 (0.012)	0.000	-0.044 (0.09)	0.041 (0.011)	0.000	-0.049 (0.09)
n=500; B=0.3; Med =5%	HDMA	0.094 (0.02)	0.001	0.004 (0.09)	0.091 (0.02)	0.001	0.001 (0.09)	0.088 (0.021)	0.001	-0.002 (0.09)
	HIMA	0.088 (0.025)	0.001	-0.002 (0.09)	0.089 (0.025)	0.001	-0.001 (0.09)	0.09 (0.025)	0.001	0 (0.09)
	MITM	0.048 (0.	0.000	-0.042 (0.	0.034 (0.	0.000	-0.056 (0.	0.027 (0.	0.000	-0.063 (0.

		p=200			p=400			p=600		
**Simulation Scenario**	**Method**	**Mean (SD)**	**SE**	**Bias (Truth)**	**Mean (SD)**	**SE**	**Bias (Truth)**	**Mean (SD)**	**SE**	**Bias (Truth)**
		012)		09)	009)		09)	007)		09)
n=1000; B=0.3; Med=5%	HDMA	0.091 (0.014)	0.000	0.001 (0.09)	0.091 (0.014)	0.000	0.001 (0.09)	0.092 (0.014)	0.000	0.002 (0.09)
	HIMA	0.092 (0.015)	0.000	0.002 (0.09)	0.092 (0.015)	0.000	0.002 (0.09)	0.094 (0.015)	0.000	0.004 (0.09)
	MITM	0.047 (0.008)	0.000	-0.043 (0.09)	0.032 (0.007)	0.000	-0.058 (0.09)	0.025 (0.006)	0.000	-0.065 (0.09)
n=200; B=0.3; Med=10%	HDMA	0.089 (0.032)	0.001	-0.001 (0.09)	0.089 (0.032)	0.001	-0.001 (0.09)	0.098 (0.036)	0.001	0.008 (0.09)
	HIMA	0.086 (0.04)	0.001	-0.004 (0.09)	0.088 (0.046)	0.001	-0.002 (0.09)	0.096 (0.055)	0.002	0.006 (0.09)

		p=200			p=400			p=600		
**Simulation Scenario**	**Method**	**Mean (SD)**	**SE**	**Bias (Truth)**	**Mean (SD)**	**SE**	**Bias (Truth)**	**Mean (SD)**	**SE**	**Bias (Truth)**
	MITM	0.042 (0.013)	0.000	-0.048 (0.09)	0.034 (0.009)	0.000	-0.056 (0.09)	0.03 (0.008)	0.000	-0.06 (0.09)
	HDMA	0.092 (0.02)	0.001	0.002 (0.09)	0.089 (0.02)	0.001	-0.001 (0.09)	0.085 (0.022)	0.001	-0.005 (0.09)
n=500; B=0.3; Med=10%	HIMA	0.09 (0.024)	0.001	0 (0.09)	0.091 (0.024)	0.001	0.001 (0.09)	0.088 (0.027)	0.001	-0.002 (0.09)
	MITM	0.033 (0.009)	0.000	-0.057 (0.09)	0.022 (0.006)	0.000	-0.068 (0.09)	0.018 (0.004)	0.000	-0.072 (0.09)
n=1000; B=0.3; Med	HDMA	0.091 (0.014)	0.000	0.001 (0.09)	0.09 (0.014)	0.000	0 (0.09)	0.09 (0.014)	0.000	0 (0.09)

		p=200			p=400			p=600		
**Simulation Scenario**	**Method**	**Mean (SD)**	**SE**	**Bias (Truth)**	**Mean (SD)**	**SE**	**Bias (Truth)**	**Mean (SD)**	**SE**	**Bias (Truth)**
=10%	HIMA	0.093 (0.015)	0.000	0.003 (0.09)	0.092 (0.015)	0.000	0.002 (0.09)	0.094 (0.015)	0.000	0.004 (0.09)
	MITM	0.032 (0.007)	0.000	-0.058 (0.09)	0.019 (0.005)	0.000	-0.071 (0.09)	0.015 (0.004)	0.000	-0.075 (0.09)

Table S2. Summary of total indirect effect (TIE) estimates and bias from the truth. The simulation scenario column describes the unique sample size (n), mediator beta value (B), percent of true mediators (Med), and independence yes/no (Ind). Bias was calculated by subtracting the truth from the mean of the simulation run.

		p=200			p=400			p=600		
**Simulation Scenario**	**Method**	**Mean (SD)**	**SE**	**Bias (Truth)**	**Mean (SD)**	**SE**	**Bias (Truth)**	**Mean (SD)**	**SE**	**Bias (Truth)**
n=200; B=0.1; Med=2%; Ind=no	HDMA	0.026 (0.027)	0.001	-0.014 (0.04)	0.048 (0.044)	0.001	-0.032 (0.08)	0.052 (0.049)	0.002	-0.068 (0.12)
	HIMA	0.005 (0.021)	0.000	-0.035 (0.04)	0.013 (0.037)	0.001	-0.067 (0.08)	0.027 (0.053)	0.001	-0.093 (0.12)
	MITM	0.035 (0.05)	0.000	-0.005 (0.04)	0.056 (0.006)	0.000	-0.024 (0.08)	0.063 (0.009)	0.000	-0.057 (0.12)
n=500; B=0.1; Med=2%; Ind=no	HDMA	0.01 (0.031)	0.001	-0.03 (0.04)	0.03 (0.022)	0.001	-0.05 (0.08)	0.05 (0.026)	0.001	-0.07 (0.12)
	HIMA	0.008 (0.009)	0.000	-0.032 (0.04)	0.01 (0.012)	0.000	-0.07 (0.08)	0.011 (0.013)	0.000	-0.109 (0.12)

		p=200			p=400			p=600		
**Simulation Scenario**	**Method**	**Mean (SD)**	**SE**	**Bias (Truth)**	**Mean (SD)**	**SE**	**Bias (Truth)**	**Mean (SD)**	**SE**	**Bias (Truth)**
	MITM	0.028 (0.008)	0.000	-0.012 (0.04)	0.031 (0.013)	0.000	-0.049 (0.08)	0.032 (0.012)	0.000	-0.088 (0.12)
n=1000; B=0.1; Med=2%; Ind=no	HDMA	0.013 (0.047)	0.001	-0.027 (0.04)	0.028 (0.024)	0.001	-0.052 (0.08)	0.052 (0.023)	0.001	-0.068 (0.12)
	HIMA	0.014 (0.01)	0.000	-0.026 (0.04)	0.021 (0.016)	0.000	-0.059 (0.08)	0.026 (0.021)	0.001	-0.094 (0.12)
	MITM	0.026 (0.012)	0.000	-0.014 (0.04)	0.04 (0.021)	0.001	-0.04 (0.08)	0.053 (0.025)	0.001	-0.067 (0.12)
n=200; B=0.1; Med=5%	HDMA	0.037 (0.034)	0.001	-0.063 (0.1)	0.072 (0.051)	0.002	-0.128 (0.2)	0.091 (0.059)	0.002	-0.209 (0.3)

		p=200			p=400			p=600		
**Simulation Scenario**	**Method**	**Mean (SD)**	**SE**	**Bias (Truth)**	**Mean (SD)**	**SE**	**Bias (Truth)**	**Mean (SD)**	**SE**	**Bias (Truth)**
; Ind=no	HIMA	0.014 (0.032)	0.001	-0.086 (0.1)	0.047 (0.059)	0.002	-0.153 (0.2)	0.071 (0.069)	0.002	-0.229 (0.3)
	MITM	0.053 (0.029)	0.000	-0.047 (0.1)	0.061 (0.022)	0.000	-0.139 (0.2)	0.053 (0.03)	0.000	-0.247 (0.3)
n=500; B=0.1; Med =5%; Ind=no	HDMA	0.026 (0.039)	0.001	-0.074 (0.1)	0.062 (0.032)	0.001	-0.138 (0.2)	0.109 (0.04)	0.001	-0.191 (0.3)
	HIMA	0.016 (0.018)	0.000	-0.084 (0.1)	0.022 (0.028)	0.001	-0.178 (0.2)	0.026 (0.04)	0.001	-0.274 (0.3)
	MITM	0.035 (0.02)	0.000	-0.065 (0.1)	0.042 (0.025)	0.001	-0.158 (0.2)	0.045 (0.027)	0.001	-0.255 (0.3)
n=1000; B=0.1; Med	HDMA	0.03 (0.057)	0.002	-0.07 (0.1)	0.075 (0.	0.001	-0.125	0.138 (0.	0.001	-0.162

		p=200			p=400			p=600		
**Simulation Scenario**	**Method**	**Mean (SD)**	**SE**	**Bias (Truth)**	**Mean (SD)**	**SE**	**Bias (Truth)**	**Mean (SD)**	**SE**	**Bias (Truth)**
=5%; Ind=no					038)		(0.2)	038)		(0.3)
	HIMA	0.035 (0.024)	0.001	-0.065 (0.1)	0.059 (0.041)	0.001	-0.141 (0.2)	0.076 (0.057)	0.002	-0.224 (0.3)
	MITM	0.061 (0.024)	0.001	-0.039 (0.1)	0.106 (0.033)	0.001	-0.094 (0.2)	0.14 (0.038)	0.001	-0.16 (0.3)
n=200; B=0.1; Med=10%; Ind=no	HDMA	0.053 (0.043)	0.001	-0.147 (0.2)	0.111 (0.06)	0.002	-0.289 (0.4)	0.146 (0.071)	0.002	-0.454 (0.6)
	HIMA	0.044 (0.057)	0.002	-0.156 (0.2)	0.102 (0.079)	0.002	-0.298 (0.4)	0.128 (0.086)	0.003	-0.472 (0.6)
	MITM	0.051 (0.033)	0.000	-0.149 (0.2)	0.049 (0.027)	0.000	-0.351 (0.4)	0.044 (0.026)	0.000	-0.556 (0.6)
n=500; B=0.	HD	0.051	0.0	-0.14	0.122	0.0	-0.27	0.197	0.0	-0.40

		p=200			p=400			p=600		
**Simulation Scenario**	**Method**	**Mean (SD)**	**SE**	**Bias (Truth)**	**Mean (SD)**	**SE**	**Bias (Truth)**	**Mean (SD)**	**SE**	**Bias (Truth)**
1; Med =10 %; Ind= no	MA	(0.046)	01	9 (0.2)	(0.047)	01	8 (0.4)	(0.056)	02	3 (0.6)
	HIMA	0.04 (0.042)	0.001	-0.16 (0.2)	0.071 (0.081)	0.002	-0.329 (0.4)	0.134 (0.129)	0.004	-0.466 (0.6)
	MITM	0.059 (0.035)	0.001	-0.141 (0.2)	0.074 (0.043)	0.001	-0.326 (0.4)	0.084 (0.045)	0.001	-0.516 (0.6)
n=1000; B=0.1; Med =10 %; Ind= no	HDMA	0.059 (0.075)	0.002	-0.141 (0.2)	0.165 (0.053)	0.002	-0.235 (0.4)	0.286 (0.056)	0.002	-0.314 (0.6)
	HIMA	0.092 (0.049)	0.002	-0.108 (0.2)	0.175 (0.093)	0.003	-0.225 (0.4)	0.276 (0.132)	0.004	-0.324 (0.6)
	MITM	0.131 (0.035)	0.001	-0.069 (0.2)	0.207 (0.042)	0.001	-0.193 (0.4)	0.259 (0.044)	0.001	-0.341 (0.6)
n=200;	HD	0.28	0.0	-0.	0.58	0.0	-0.	0.80	0.0	-0.

		p=200			p=400			p=600		
**Simulation Scenario**	**Method**	**Mean (SD)**	**SE**	**Bias (Truth)**	**Mean (SD)**	**SE**	**Bias (Truth)**	**Mean (SD)**	**SE**	**Bias (Truth)**
B=0.3; Med=2%; Ind=no	MA	3 (0.083)	03	077 (0.36)	6 (0.121)	04	134 (0.72)	1 (0.16)	05	279 (1.08)
	HIMA	0.271 (0.105)	0.003	-0.089 (0.36)	0.557 (0.159)	0.005	-0.163 (0.72)	0.765 (0.19)	0.006	-0.315 (1.08)
	MITM	0.222 (0.076)	0.002	-0.138 (0.36)	0.298 (0.104)	0.003	-0.422 (0.72)	0.316 (0.108)	0.003	-0.764 (1.08)
n=500; B=0.3; Med=2%; Ind=no	HDMA	0.274 (0.108)	0.003	-0.086 (0.36)	0.615 (0.064)	0.002	-0.105 (0.72)	0.934 (0.074)	0.002	-0.146 (1.08)
	HIMA	0.341 (0.051)	0.002	-0.019 (0.36)	0.687 (0.081)	0.003	-0.033 (0.72)	1.032 (0.098)	0.003	-0.048 (1.08)

		p=200			p=400			p=600		
**Simulation Scenario**	**Method**	**Mean (SD)**	**SE**	**Bias (Truth)**	**Mean (SD)**	**SE**	**Bias (Truth)**	**Mean (SD)**	**SE**	**Bias (Truth)**
	MITM	0.262 (0.033)	0.001	-0.098 (0.36)	0.418 (0.04)	0.001	-0.302 (0.72)	0.51 (0.043)	0.001	-0.57 (1.08)
n=1000; B=0.3; Med=2%; Ind=no	HDMA	0.211 (0.155)	0.005	-0.149 (0.36)	0.664 (0.064)	0.002	-0.056 (0.72)	0.966 (0.055)	0.002	-0.114 (1.08)
	HIMA	0.372 (0.031)	0.001	0.012 (0.36)	0.74 (0.049)	0.002	0.02 (0.72)	1.112 (0.059)	0.002	0.032 (1.08)
	MITM	0.264 (0.026)	0.001	-0.096 (0.36)	0.418 (0.03)	0.001	-0.302 (0.72)	0.52 (0.027)	0.001	-0.56 (1.08)
n=200; B=0.3; Med=5%	HDMA	0.68 (0.148)	0.005	-0.22 (0.9)	1.168 (0.237)	0.008	-0.632 (1.8)	1.371 (0.278)	0.009	-1.329 (2.7)

		p=200			p=400			p=600		
**Simulation Scenario**	**Method**	**Mean (SD)**	**SE**	**Bias (Truth)**	**Mean (SD)**	**SE**	**Bias (Truth)**	**Mean (SD)**	**SE**	**Bias (Truth)**
; Ind=no	HIMA	0.741 (0.176)	0.006	-0.159 (0.9)	1.157 (0.284)	0.009	-0.643 (1.8)	1.279 (0.314)	0.010	-1.421 (2.7)
	MITM	0.385 (0.101)	0.003	-0.515 (0.9)	0.407 (0.114)	0.004	-1.393 (1.8)	0.332 (0.118)	0.004	-2.368 (2.7)
n=500; B=0.3; Med=5%; Ind=no	HDMA	0.723 (0.169)	0.005	-0.177 (0.9)	1.557 (0.122)	0.004	-0.243 (1.8)	2.204 (0.188)	0.006	-0.496 (2.7)
	HIMA	0.888 (0.084)	0.003	-0.012 (0.9)	1.749 (0.153)	0.005	-0.051 (1.8)	2.475 (0.237)	0.007	-0.225 (2.7)
	MITM	0.467 (0.044)	0.001	-0.433 (0.9)	0.621 (0.046)	0.001	-1.179 (1.8)	0.671 (0.051)	0.002	-2.029 (2.7)
n=1000; B=0.3;	HDMA	0.497 (0.	0.008	-0.403	1.718 (0.	0.003	-0.082	2.488 (0.	0.003	-0.212

		p=200			p=400			p=600		
**Simulation Scenario**	**Method**	**Mean (SD)**	**SE**	**Bias (Truth)**	**Mean (SD)**	**SE**	**Bias (Truth)**	**Mean (SD)**	**SE**	**Bias (Truth)**
Med=5%; Ind=no		24)		(0.9)	103)		(1.8)	099)		(2.7)
	HIMA	0.931 (0.051)	0.002	0.031 (0.9)	1.858 (0.084)	0.003	0.058 (1.8)	2.77 (0.117)	0.004	0.07 (2.7)
	MITM	0.472 (0.034)	0.001	-0.428 (0.9)	0.641 (0.029)	0.001	-1.159 (1.8)	0.724 (0.026)	0.001	-1.976 (2.7)
n=200; B=0.3; Med=10%; Ind=no	HDMA	1.141 (0.245)	0.008	-0.659 (1.8)	1.603 (0.323)	0.010	-1.997 (3.6)	1.768 (0.369)	0.012	-3.632 (5.4)
	HIMA	1.267 (0.286)	0.009	-0.533 (1.8)	1.595 (0.364)	0.011	-2.005 (3.6)	1.649 (0.398)	0.013	-3.751 (5.4)
	MITM	0.495 (0.102)	0.003	-1.305 (1.8)	0.413 (0.117)	0.004	-3.187 (3.6)	0.287 (0.116)	0.004	-5.113 (5.4)
n=500; B=0.	HD	1.46 (0.	0.0	-0.34	2.757	0.0	-0.84	3.37 (0.	0.0	-2.03

		p=200			p=400			p=600		
**Simulation Scenario**	**Method**	**Mean (SD)**	**SE**	**Bias (Truth)**	**Mean (SD)**	**SE**	**Bias (Truth)**	**Mean (SD)**	**SE**	**Bias (Truth)**
3; Med=10%; Ind=no	MA	248)	08	(1.8)	(0.268)	08	3 (3.6)	351)	11	(5.4)
	HIMA	1.788 (0.147)	0.005	-0.012 (1.8)	3.1 (0.328)	0.010	-0.5 (3.6)	3.738 (0.439)	0.014	-1.662 (5.4)
	MITM	0.623 (0.05)	0.002	-1.177 (1.8)	0.715 (0.046)	0.001	-2.885 (3.6)	0.698 (0.055)	0.002	-4.702 (5.4)
n=1000; B=0.3; Med=10%; Ind=no	HDMA	0.995 (0.351)	0.011	-0.805 (1.8)	3.409 (0.177)	0.006	-0.191 (3.6)	4.625 (0.26)	0.008	-0.775 (5.4)
	HIMA	1.871 (0.08)	0.003	0.071 (1.8)	3.65 (0.173)	0.005	0.05 (3.6)	5.127 (0.301)	0.010	-0.273 (5.4)
	MITM	0.641 (0.036)	0.001	-1.159 (1.8)	0.77 (0.029)	0.001	-2.83 (3.6)	0.815 (0.026)	0.001	-4.585 (5.4)
n=200;	HD	0.08	0.0	0.04	0.05	0.0	-0.	0.06	0.0	-0.

		p=200			p=400			p=600		
**Simulation Scenario**	**Method**	**Mean (SD)**	**SE**	**Bias (Truth)**	**Mean (SD)**	**SE**	**Bias (Truth)**	**Mean (SD)**	**SE**	**Bias (Truth)**
B=0.1; Med=2%; Ind=yes	MA	6 (0.057)	02	6 (0.04)	6 (0.055)	02	024 (0.08)	4 (0.054)	02	056 (0.12)
	HIMA	0.005 (0.015)	0.000	-0.035 (0.04)	0.013 (0.042)	0.001	-0.067 (0.08)	0.035 (0.06)	0.002	-0.085 (0.12)
	MITM	0.059 (0.012)	0.000	0.019 (0.04)	0.059 (0.01)	0.000	-0.021 (0.08)	0.068 (0.007)	0.000	-0.052 (0.12)
n=500; B=0.1; Med=2%; Ind=yes	HDMA	0.067 (0.025)	0.001	0.027 (0.04)	0.105 (0.042)	0.001	0.025 (0.08)	0.088 (0.038)	0.001	-0.032 (0.12)
	HIMA	0.007 (0.008)	0.000	-0.033 (0.04)	0.008 (0.009)	0.000	-0.072 (0.08)	0.009 (0.012)	0.000	-0.111 (0.12)

		p=200			p=400			p=600		
**Simulation Scenario**	**Method**	**Mean (SD)**	**SE**	**Bias (Truth)**	**Mean (SD)**	**SE**	**Bias (Truth)**	**Mean (SD)**	**SE**	**Bias (Truth)**
	MITM	0.028 (0.009)	0.000	-0.012 (0.04)	0.029 (0.009)	0.000	-0.051 (0.08)	0.032 (0.014)	0.000	-0.088 (0.12)
n=1000; B=0.1; Med=2%; Ind=yes	HDMA	0.052 (0.013)	0.000	0.012 (0.04)	0.099 (0.019)	0.001	0.019 (0.08)	0.139 (0.024)	0.001	0.019 (0.12)
	HIMA	0.013 (0.01)	0.000	-0.027 (0.04)	0.02 (0.015)	0.000	-0.06 (0.08)	0.027 (0.02)	0.001	-0.093 (0.12)
	MITM	0.026 (0.012)	0.000	-0.014 (0.04)	0.037 (0.019)	0.001	-0.043 (0.08)	0.049 (0.023)	0.001	-0.071 (0.12)
n=200; B=0.1; Med=5%	HDMA	0.118 (0.067)	0.002	0.018 (0.1)	0.097 (0.062)	0.002	-0.103 (0.2)	0.116 (0.066)	0.002	-0.184 (0.3)

		p=200			p=400			p=600		
**Simulation Scenario**	**Method**	**Mean (SD)**	**SE**	**Bias (Truth)**	**Mean (SD)**	**SE**	**Bias (Truth)**	**Mean (SD)**	**SE**	**Bias (Truth)**
; Ind=yes	HIMA	0.01 (0.023)	0.001	-0.09 (0.1)	0.04 (0.058)	0.002	-0.16 (0.2)	0.082 (0.077)	0.002	-0.218 (0.3)
	MITM	0.053 (0.007)	0.000	-0.047 (0.1)	0.054 (0.012)	0.000	-0.146 (0.2)	0.063 (0.015)	0.000	-0.237 (0.3)
n=500; B=0.1; Med=5%; Ind=yes	HDMA	0.122 (0.031)	0.001	0.022 (0.1)	0.175 (0.049)	0.002	-0.025 (0.2)	0.194 (0.047)	0.001	-0.106 (0.3)
	HIMA	0.014 (0.015)	0.000	-0.086 (0.1)	0.014 (0.017)	0.000	-0.186 (0.2)	0.017 (0.026)	0.001	-0.283 (0.3)
	MITM	0.034 (0.017)	0.000	-0.066 (0.1)	0.035 (0.018)	0.000	-0.165 (0.2)	0.038 (0.021)	0.000	-0.262 (0.3)
n=1000; B=0.1; Med	HDMA	0.109 (0.	0.001	0.009 (0.1)	0.207 (0.	0.001	0.007 (0.2)	0.291 (0.	0.001	-0.009

		p=200			p=400			p=600		
**Simulation Scenario**	**Method**	**Mean (SD)**	**SE**	**Bias (Truth)**	**Mean (SD)**	**SE**	**Bias (Truth)**	**Mean (SD)**	**SE**	**Bias (Truth)**
=5%; Ind=yes		018)			027)			035)		(0.3)
	HIMA	0.035 (0.022)	0.001	-0.065 (0.1)	0.057 (0.038)	0.001	-0.143 (0.2)	0.073 (0.054)	0.002	-0.227 (0.3)
	MITM	0.058 (0.022)	0.001	-0.042 (0.1)	0.095 (0.031)	0.001	-0.105 (0.2)	0.124 (0.036)	0.001	-0.176 (0.3)
n=200; B=0.1; Med=10%; Ind=yes	HDMA	0.161 (0.08)	0.003	-0.039 (0.2)	0.162 (0.071)	0.002	-0.238 (0.4)	0.192 (0.08)	0.003	-0.408 (0.6)
	HIMA	0.02 (0.037)	0.001	-0.18 (0.2)	0.095 (0.087)	0.002	-0.305 (0.4)	0.152 (0.09)	0.003	-0.448 (0.6)
	MITM	0.054 (0.024)	0.000	-0.146 (0.2)	0.057 (0.015)	0.000	-0.343 (0.4)	0.052 (0.019)	0.000	-0.548 (0.6)
n=500; B=0.	HD	0.196	0.0	-0.00	0.287	0.0	-0.11	0.351	0.0	-0.24

		p=200			p=400			p=600		
Simulation Scenario	Method	Mean (SD)	SE	Bias (Truth)	Mean (SD)	SE	Bias (Truth)	Mean (SD)	SE	Bias (Truth)
1; Med=10%; Ind=yes	MA	(0.038)	01	4 (0.2)	(0.056)	02	3 (0.4)	(0.066)	02	9 (0.6)
	HIMA	0.024 (0.028)	0.001	-0.176 (0.2)	0.026 (0.039)	0.001	-0.374 (0.4)	0.026 (0.055)	0.001	-0.574 (0.6)
	MITM	0.047 (0.027)	0.001	-0.153 (0.2)	0.053 (0.032)	0.001	-0.347 (0.4)	0.053 (0.033)	0.001	-0.547 (0.6)
n=1000; B=0.1; Med=10%; Ind=yes	HDMA	0.201 (0.025)	0.001	0.001 (0.2)	0.381 (0.038)	0.001	-0.019 (0.4)	0.532 (0.047)	0.001	-0.068 (0.6)
	HIMA	0.08 (0.046)	0.001	-0.12 (0.2)	0.13 (0.085)	0.003	-0.27 (0.4)	0.155 (0.133)	0.004	-0.445 (0.6)
	MITM	0.119 (0.029)	0.001	-0.081 (0.2)	0.182 (0.036)	0.001	-0.218 (0.4)	0.217 (0.04)	0.001	-0.383 (0.6)
n=200;	HD	0.41	0.0	0.05	0.66	0.0	-0.	0.87	0.0	-0.

		p=200			p=400			p=600		
**Simulation Scenario**	**Method**	**Mean (SD)**	**SE**	**Bias (Truth)**	**Mean (SD)**	**SE**	**Bias (Truth)**	**Mean (SD)**	**SE**	**Bias (Truth)**
B=0.3; Med=2%; Ind=yes	MA	4 (0.084)	03	4 (0.36)	6 (0.127)	04	054 (0.72)	7 (0.167)	05	203 (1.08)
	HIMA	0.246 (0.1)	0.003	-0.114 (0.36)	0.572 (0.147)	0.005	-0.148 (0.72)	0.821 (0.188)	0.006	-0.259 (1.08)
	MITM	0.214 (0.073)	0.002	-0.146 (0.36)	0.293 (0.095)	0.003	-0.427 (0.72)	0.296 (0.106)	0.003	-0.784 (1.08)
n=500; B=0.3; Med=2%; Ind=yes	HDMA	0.396 (0.044)	0.001	0.036 (0.36)	0.764 (0.064)	0.002	0.044 (0.72)	1.085 (0.088)	0.003	0.005 (1.08)
	HIMA	0.337 (0.052)	0.002	-0.023 (0.36)	0.668 (0.078)	0.002	-0.052 (0.72)	1.017 (0.102)	0.003	-0.063 (1.08)
	MITM	0.265	0.0	-0.09	0.417	0.0	-0.30	0.51 (0.	0.0	-0.57

Simulation Scenario	Method	p=200 Mean (SD)	p=200 SE	p=200 Bias (Truth)	p=400 Mean (SD)	p=400 SE	p=400 Bias (Truth)	p=600 Mean (SD)	p=600 SE	p=600 Bias (Truth)
		(0.029)	01	5 (0.36)	(0.035)	01	3 (0.72)	039)	01	(1.08)
n=1000; B=0.3; Med=2%; Ind=yes	HDMA	0.378 (0.029)	0.001	0.018 (0.36)	0.747 (0.043)	0.001	0.027 (0.72)	1.115 (0.052)	0.002	0.035 (1.08)
	HIMA	0.371 (0.031)	0.001	0.011 (0.36)	0.736 (0.048)	0.002	0.016 (0.72)	1.105 (0.063)	0.002	0.025 (1.08)
	MITM	0.265 (0.02)	0.001	-0.095 (0.36)	0.417 (0.023)	0.001	-0.303 (0.72)	0.519 (0.022)	0.001	-0.561 (1.08)
n=200; B=0.3; Med=5%; Ind=yes	HDMA	0.922 (0.124)	0.004	0.022 (0.9)	1.451 (0.219)	0.007	-0.349 (1.8)	1.693 (0.301)	0.010	-1.007 (2.7)
	HIMA	0.754 (0.179)	0.006	-0.146 (0.9)	1.35 (0.258)	0.008	-0.45 (1.8)	1.538 (0.333)	0.011	-1.162 (2.7)

		p=200			p=400			p=600		
**Simulation Scenario**	**Method**	**Mean (SD)**	**SE**	**Bias (Truth)**	**Mean (SD)**	**SE**	**Bias (Truth)**	**Mean (SD)**	**SE**	**Bias (Truth)**
	MITM	0.359 (0.095)	0.003	-0.541 (0.9)	0.338 (0.109)	0.003	-1.462 (1.8)	0.251 (0.106)	0.003	-2.449 (2.7)
n=500; B=0.3; Med=5%; Ind=yes	HDMA	0.943 (0.064)	0.002	0.043 (0.9)	1.82 (0.094)	0.003	0.02 (1.8)	2.613 (0.139)	0.004	-0.087 (2.7)
	HIMA	0.875 (0.082)	0.003	-0.025 (0.9)	1.764 (0.126)	0.004	-0.036 (1.8)	2.65 (0.176)	0.006	-0.05 (2.7)
	MITM	0.472 (0.034)	0.001	-0.428 (0.9)	0.62 (0.041)	0.001	-1.18 (1.8)	0.653 (0.051)	0.002	-2.047 (2.7)
n=1000; B=0.3; Med=5%; Ind=yes	HDMA	0.924 (0.045)	0.001	0.024 (0.9)	1.829 (0.066)	0.002	0.029 (1.8)	2.724 (0.082)	0.003	0.024 (2.7)
	HIMA	0.93 (0.051)	0.002	0.03 (0.9)	1.849 (0.	0.003	0.049 (1.8)	2.783 (0.	0.003	0.083 (2.7)

		p=200			p=400			p=600		
**Simulation Scenario**	**Method**	**Mean (SD)**	**SE**	**Bias (Truth)**	**Mean (SD)**	**SE**	**Bias (Truth)**	**Mean (SD)**	**SE**	**Bias (Truth)**
					082)			11)		
	MITM	0.474 (0.023)	0.001	-0.426 (0.9)	0.642 (0.022)	0.001	-1.158 (1.8)	0.727 (0.022)	0.001	-1.973 (2.7)
n=200; B=0.3; Med=10%; Ind=yes	HDMA	1.659 (0.194)	0.006	-0.141 (1.8)	2.214 (0.335)	0.011	-1.386 (3.6)	2.291 (0.417)	0.013	-3.109 (5.4)
	HIMA	1.545 (0.248)	0.008	-0.255 (1.8)	1.999 (0.377)	0.012	-1.601 (3.6)	1.97 (0.433)	0.014	-3.43 (5.4)
	MITM	0.418 (0.103)	0.003	-1.382 (1.8)	0.27 (0.108)	0.003	-3.33 (3.6)	0.167 (0.089)	0.003	-5.233 (5.4)
n=500; B=0.3; Med=10	HDMA	1.833 (0.092)	0.003	0.033 (1.8)	3.484 (0.165)	0.005	-0.116 (3.6)	4.644 (0.294)	0.009	-0.756 (5.4)

		p=200			p=400			p=600		
**Simulation Scenario**	**Method**	**Mean (SD)**	**SE**	**Bias (Truth)**	**Mean (SD)**	**SE**	**Bias (Truth)**	**Mean (SD)**	**SE**	**Bias (Truth)**
%; Ind=yes	HIMA	1.785 (0.122)	0.004	-0.015 (1.8)	3.557 (0.214)	0.007	-0.043 (3.6)	4.789 (0.371)	0.012	-0.611 (5.4)
	MITM	0.629 (0.037)	0.001	-1.171 (1.8)	0.685 (0.051)	0.002	-2.915 (3.6)	0.609 (0.064)	0.002	-4.791 (5.4)
n=1000; B=0.3; Med=10%; Ind=yes	HDMA	1.827 (0.063)	0.002	0.027 (1.8)	3.611 (0.091)	0.003	0.011 (3.6)	5.35 (0.123)	0.004	-0.05 (5.4)
	HIMA	1.863 (0.078)	0.002	0.063 (1.8)	3.715 (0.124)	0.004	0.115 (3.6)	5.571 (0.179)	0.006	0.171 (5.4)
	MITM	0.643 (0.022)	0.001	-1.157 (1.8)	0.777 (0.02)	0.001	-2.823 (3.6)	0.814 (0.024)	0.001	-4.586 (5.4)

Table S3. Sensitivity and specificity summaries. The simulation scenario column describes the unique sample size (n), mediator beta value (B), percent of true mediators (Med), and independence yes/no (Ind).

		p=200		p=400		p=600	
**Simulation Scenario**	**Method**	**Sensitivity (SD)**	**Specificity (SD)**	**Sensitivity (SD)**	**Specificity (SD)**	**Sensitivity (SD)**	**Specificity (SD)**
n=200; B=0.1; Med=2%; Ind=no	HDMA	0.38 (0.24)	0.95 (0.02)	0.26 (0.15)	0.95 (0.01)	0.21 (0.11)	0.96 (0.01)
	HIMA	0.12 (0.16)	0.99 (0.02)	0.16 (0.17)	0.97 (0.04)	0.21 (0.14)	0.95 (0.02)
	MITM	0.25 (0)	1 (0)	0.08 (0.06)	1 (0)	0.08 (0)	1 (0)
n=500; B=0.1; Med=2%; Ind=no	HDMA	0.75 (0.21)	0.95 (0.02)	0.67 (0.16)	0.95 (0.01)	0.55 (0.15)	0.95 (0.01)
	HIMA	0.27 (0.2)	1 (0.01)	0.17 (0.13)	1 (0)	0.13 (0.11)	1 (0)
	MITM	0.28 (0.08)	1 (0)	0.14 (0.05)	1 (0)	0.1 (0.04)	1 (0)
n=1000; B=0.1; Med=2%; Ind=no	HDMA	0.94 (0.11)	0.95 (0.02)	0.92 (0.09)	0.95 (0.01)	0.91 (0.08)	0.95 (0.01)
	HIMA	0.53 (0.27)	1 (0.01)	0.44 (0.26)	1 (0)	0.4 (0.24)	1 (0)
	MITM	0.45 (0.21)	1 (0)	0.33 (0.17)	1 (0)	0.3 (0.15)	1 (0)
n=200; B=0.1; Med=5%; Ind=no	HDMA	0.34 (0.15)	0.95 (0.02)	0.23 (0.1)	0.96 (0.01)	0.19 (0.07)	0.96 (0.01)
	HIMA	0.1 (0.12)	0.99 (0.03)	0.17 (0.14)	0.96 (0.04)	0.19 (0.09)	0.95 (0.02)
	MITM	0.1 (0)	1 (0)	0.04 (0.02)	1 (0)	0.04 (0.01)	1 (0)
n=500; B=0.1; Med=5%; Ind=no	HDMA	0.71 (0.13)	0.95 (0.02)	0.6 (0.11)	0.95 (0.01)	0.53 (0.09)	0.95 (0.01)
	HIMA	0.2 (0.16)	1 (0.01)	0.12 (0.1)	1 (0)	0.09 (0.09)	1 (0.01)
	MITM	0.15 (0.08)	1 (0)	0.08 (0.04)	1 (0)	0.06 (0.03)	1 (0)
n=1000; B=0.1; Med=5%; Ind=no	HDMA	0.92 (0.08)	0.95 (0.02)	0.89 (0.07)	0.95 (0.01)	0.87 (0.06)	0.95 (0.01)
	HIMA	0.55 (0.28)	0.99 (0.01)	0.48 (0.26)	0.99 (0.01)	0.41 (0.25)	0.99 (0.01)
	MITM	0.46 (0.18)	1 (0)	0.4 (0.13)	1 (0)	0.36 (0.11)	1 (0)
n=200; B=0.1;	HDMA	0.31 (0.1)	0.95 (0.02)	0.21 (0.06)	0.96 (0.01)	0.16 (0.05)	0.97 (0.01)
	HIMA	0.09 (0.1)	0.99 (0.03)	0.18 (0.12)	0.95 (0.04)	0.17 (0.06)	0.95 (0.01)

		p=200		p=400		p=600	
Simulation Scenario	Method	Sensitivity (SD)	Specificity (SD)	Sensitivity (SD)	Specificity (SD)	Sensitivity (SD)	Specificity (SD)
Med=10%; Ind=no	MITM	0.05 (0.02)	1 (0)	0.03 (0.01)	1 (0)	0.02 (0)	1 (0)
n=500; B=0.1; Med=10%; Ind=no	HDMA	0.64 (0.1)	0.95 (0.02)	0.54 (0.08)	0.95 (0.01)	0.47 (0.07)	0.95 (0.01)
	HIMA	0.17 (0.16)	1 (0.01)	0.09 (0.11)	1 (0.01)	0.06 (0.09)	1 (0.01)
	MITM	0.12 (0.07)	1 (0)	0.07 (0.04)	1 (0)	0.05 (0.03)	1 (0)
n=1000; B=0.1; Med=10%; Ind=no	HDMA	0.89 (0.07)	0.95 (0.02)	0.85 (0.06)	0.95 (0.01)	0.83 (0.05)	0.95 (0.01)
	HIMA	0.6 (0.27)	0.98 (0.02)	0.5 (0.27)	0.99 (0.01)	0.4 (0.29)	0.99 (0.01)
	MITM	0.54 (0.13)	1 (0)	0.46 (0.1)	1 (0)	0.39 (0.08)	1 (0)
n=200; B=0.3; Med=2%; Ind=no	HDMA	0.99 (0.05)	0.95 (0.02)	0.96 (0.07)	0.96 (0.01)	0.89 (0.09)	0.97 (0.01)
	HIMA	0.88 (0.2)	0.98 (0.03)	0.92 (0.12)	0.96 (0.03)	0.87 (0.11)	0.96 (0.01)
	MITM	0.71 (0.24)	1 (0)	0.57 (0.19)	1 (0)	0.43 (0.17)	1 (0)
n=500; B=0.3; Med=2%; Ind=no	HDMA	1 (0)	0.95 (0.02)	1 (0)	0.95 (0.01)	1 (0)	0.96 (0.01)
	HIMA	1 (0.01)	1 (0.01)	1 (0.01)	1 (0.01)	1 (0)	0.99 (0.01)
	MITM	1 (0.02)	1 (0)	0.99 (0.04)	1 (0)	0.97 (0.05)	1 (0)
n=1000; B=0.3; Med=2%; Ind=no	HDMA	1 (0)	0.95 (0.02)	1 (0)	0.95 (0.01)	1 (0)	0.95 (0.01)
	HIMA	1 (0)	1 (0)	1 (0)	1 (0)	1 (0)	1 (0)
	MITM	1 (0)	1 (0)	1 (0)	1 (0)	1 (0)	1 (0)
n=200; B=0.3; Med=5%; Ind=no	HDMA	0.98 (0.05)	0.96 (0.01)	0.85 (0.08)	0.97 (0.01)	0.66 (0.09)	0.98 (0.01)
	HIMA	0.93 (0.12)	0.95 (0.03)	0.82 (0.1)	0.96 (0.02)	0.62 (0.1)	0.97 (0.01)
	MITM	0.64 (0.17)	1 (0)	0.38 (0.13)	1 (0)	0.21 (0.1)	1 (0)
n=500; B=0.3; Med=5%; Ind=no	HDMA	1 (0)	0.95 (0.02)	1 (0.01)	0.96 (0.01)	0.99 (0.02)	0.97 (0.01)
	HIMA	1 (0)	0.99 (0.01)	1 (0.01)	0.99 (0.01)	0.99 (0.02)	0.99 (0.01)
	MITM	0.99 (0.03)	1 (0)	0.93 (0.06)	1 (0)	0.82 (0.07)	1 (0)

		p=200		p=400		p=600	
**Simulation Scenario**	**Method**	**Sensitivity (SD)**	**Specificity (SD)**	**Sensitivity (SD)**	**Specificity (SD)**	**Sensitivity (SD)**	**Specificity (SD)**
n=1000; B=0.3; Med=5%; Ind=no	HDMA	1 (0)	0.95 (0.02)	1 (0)	0.95 (0.01)	1 (0)	0.96 (0.01)
	HIMA	1 (0)	1 (0)	1 (0)	1 (0)	1 (0)	1 (0)
	MITM	1 (0)	1 (0)	1 (0.01)	1 (0)	0.99 (0.01)	1 (0)
n=200; B=0.3; Med=10%; Ind=no	HDMA	0.92 (0.06)	0.96 (0.02)	0.62 (0.08)	0.97 (0.01)	0.39 (0.06)	0.98 (0.01)
	HIMA	0.9 (0.08)	0.94 (0.03)	0.57 (0.09)	0.96 (0.01)	0.35 (0.06)	0.97 (0.01)
	MITM	0.5 (0.13)	1 (0)	0.2 (0.09)	1 (0)	0.1 (0.05)	1 (0)
n=500; B=0.3; Med=10%; Ind=no	HDMA	1 (0)	0.96 (0.01)	0.99 (0.02)	0.97 (0.01)	0.91 (0.03)	0.97 (0.01)
	HIMA	1 (0)	0.99 (0.01)	0.98 (0.02)	0.99 (0.01)	0.91 (0.03)	0.98 (0.01)
	MITM	0.96 (0.05)	1 (0)	0.78 (0.07)	1 (0)	0.58 (0.08)	1 (0)
n=1000; B=0.3; Med=10%; Ind=no	HDMA	1 (0)	0.95 (0.01)	1 (0)	0.96 (0.01)	1 (0.01)	0.97 (0.01)
	HIMA	1 (0)	1 (0)	1 (0)	1 (0)	1 (0.01)	1 (0)
	MITM	1 (0)	1 (0)	0.98 (0.02)	1 (0)	0.93 (0.03)	1 (0)
n=200; B=0.1; Med=2%; Ind=yes	HDMA	0.13 (0.16)	0.99 (0.01)	0.17 (0.13)	0.98 (0.01)	0.15 (0.1)	0.98 (0.01)
	HIMA	0.12 (0.17)	0.99 (0.02)	0.14 (0.14)	0.97 (0.03)	0.16 (0.12)	0.96 (0.02)
	MITM	0.19 (0.12)	1 (0)	0.12 (0)	1 (0)	0.08 (0)	1 (0)
n=500; B=0.1; Med=2%; Ind=yes	HDMA	0.1 (0.15)	0.97 (0.02)	0.23 (0.16)	0.99 (0.01)	0.29 (0.13)	0.98 (0.01)
	HIMA	0.26 (0.2)	1 (0.01)	0.18 (0.16)	1 (0)	0.14 (0.12)	1 (0)
	MITM	0.27 (0.07)	1 (0)	0.15 (0.06)	1 (0)	0.1 (0.04)	1 (0)
n=1000; B=0.1; Med=2%; Ind=yes	HDMA	0.08 (0.14)	0.96 (0.04)	0.23 (0.15)	0.98 (0.01)	0.38 (0.15)	0.98 (0.01)
	HIMA	0.53 (0.27)	1 (0.01)	0.43 (0.25)	1 (0)	0.37 (0.24)	1 (0)
	MITM	0.47 (0.21)	1 (0)	0.36 (0.18)	1 (0)	0.32 (0.15)	1 (0)
	HDMA	0.12 (0.1)	0.99 (0.01)	0.15 (0.08)	0.98 (0.01)	0.14 (0.06)	0.98 (0.01)

		p=200		p=400		p=600	
**Simulation Scenario**	**Method**	**Sensitivity (SD)**	**Specificity (SD)**	**Sensitivity (SD)**	**Specificity (SD)**	**Sensitivity (SD)**	**Specificity (SD)**
n=200; B=0.1; Med=5%; Ind=yes	HIMA	0.12 (0.13)	0.98 (0.03)	0.16 (0.12)	0.96 (0.03)	0.16 (0.08)	0.96 (0.02)
	MITM	0.1 (0.05)	1 (0)	0.05 (0.01)	1 (0)	0.03 (0.01)	1 (0)
n=500; B=0.1; Med=5%; Ind=yes	HDMA	0.1 (0.1)	0.97 (0.01)	0.22 (0.1)	0.98 (0.01)	0.28 (0.09)	0.98 (0.01)
	HIMA	0.21 (0.17)	0.99 (0.01)	0.15 (0.13)	1 (0.01)	0.11 (0.13)	1 (0.01)
	MITM	0.16 (0.09)	1 (0)	0.1 (0.06)	1 (0)	0.07 (0.04)	1 (0)
n=1000; B=0.1; Med=5%; Ind=yes	HDMA	0.07 (0.09)	0.96 (0.04)	0.23 (0.1)	0.98 (0.01)	0.41 (0.1)	0.98 (0.01)
	HIMA	0.49 (0.27)	0.99 (0.01)	0.44 (0.24)	0.99 (0.01)	0.39 (0.23)	0.99 (0.01)
	MITM	0.49 (0.18)	1 (0)	0.45 (0.14)	1 (0)	0.41 (0.11)	1 (0)
n=200; B=0.1; Med=10%; Ind=yes	HDMA	0.11 (0.07)	0.98 (0.01)	0.13 (0.05)	0.98 (0.01)	0.12 (0.04)	0.98 (0.01)
	HIMA	0.15 (0.13)	0.96 (0.04)	0.17 (0.08)	0.95 (0.02)	0.14 (0.05)	0.96 (0.01)
	MITM	0.05 (0.03)	1 (0)	0.03 (0.01)	1 (0)	0.02 (0.01)	1 (0)
n=500; B=0.1; Med=10%; Ind=yes	HDMA	0.09 (0.07)	0.97 (0.02)	0.22 (0.08)	0.98 (0.01)	0.25 (0.06)	0.98 (0.01)
	HIMA	0.22 (0.17)	0.98 (0.02)	0.19 (0.17)	0.98 (0.03)	0.21 (0.17)	0.97 (0.03)
	MITM	0.15 (0.09)	1 (0)	0.1 (0.06)	1 (0)	0.08 (0.05)	1 (0)
n=1000; B=0.1; Med=10%; Ind=yes	HDMA	0.07 (0.06)	0.96 (0.04)	0.25 (0.07)	0.98 (0.01)	0.41 (0.07)	0.98 (0.01)
	HIMA	0.56 (0.22)	0.97 (0.03)	0.53 (0.2)	0.96 (0.03)	0.52 (0.19)	0.96 (0.03)
	MITM	0.58 (0.13)	1 (0)	0.51 (0.1)	1 (0)	0.46 (0.08)	1 (0)
n=200; B=0.3; Med=2%; Ind=yes	HDMA	0.86 (0.18)	0.98 (0.01)	0.9 (0.11)	0.98 (0.01)	0.83 (0.11)	0.98 (0.01)
	HIMA	0.89 (0.2)	0.97 (0.03)	0.87 (0.14)	0.97 (0.02)	0.8 (0.13)	0.97 (0.01)
	MITM	0.74 (0.23)	1 (0)	0.58 (0.2)	1 (0)	0.46 (0.16)	1 (0)
n=500; B=0.3;	HDMA	0.7 (0.24)	0.97 (0.02)	1 (0.02)	0.98 (0.01)	1 (0.02)	0.98 (0.01)
	HIMA	1 (0.01)	1 (0.01)	1 (0.01)	0.99 (0.01)	1 (0.02)	0.99 (0.01)

		p=200		p=400		p=600	
Simulation Scenario	Method	Sensitivity (SD)	Specificity (SD)	Sensitivity (SD)	Specificity (SD)	Sensitivity (SD)	Specificity (SD)
Med=2%; Ind=yes	MITM	1 (0.03)	1 (0)	0.99 (0.04)	1 (0)	0.96 (0.06)	1 (0)
n=1000; B=0.3; Med=2%; Ind=yes	HDMA	0.35 (0.24)	0.96 (0.04)	0.99 (0.03)	0.97 (0.01)	1 (0)	0.98 (0.01)
	HIMA	1 (0)	1 (0)	1 (0)	1 (0)	1 (0)	1 (0)
	MITM	1 (0)	1 (0)	1 (0)	1 (0)	1 (0.01)	1 (0)
n=200; B=0.3; Med=5%; Ind=yes	HDMA	0.82 (0.14)	0.98 (0.01)	0.71 (0.11)	0.98 (0.01)	0.54 (0.09)	0.98 (0.01)
	HIMA	0.87 (0.14)	0.96 (0.02)	0.69 (0.12)	0.96 (0.01)	0.5 (0.1)	0.97 (0.01)
	MITM	0.67 (0.17)	1 (0)	0.46 (0.14)	1 (0)	0.28 (0.11)	1 (0)
n=500; B=0.3; Med=5%; Ind=yes	HDMA	0.72 (0.15)	0.97 (0.02)	0.98 (0.03)	0.98 (0.01)	0.94 (0.04)	0.98 (0.01)
	HIMA	1 (0.01)	0.99 (0.01)	0.98 (0.03)	0.99 (0.01)	0.94 (0.04)	0.99 (0.01)
	MITM	0.98 (0.05)	1 (0)	0.92 (0.06)	1 (0)	0.83 (0.07)	1 (0)
n=1000; B=0.3; Med=5%; Ind=yes	HDMA	0.33 (0.15)	0.96 (0.04)	0.99 (0.02)	0.97 (0.01)	1 (0.01)	0.98 (0.01)
	HIMA	1 (0)	1 (0)	1 (0)	1 (0)	1 (0.01)	1 (0)
	MITM	1 (0.02)	1 (0.01)	0.99 (0.02)	1 (0)	0.98 (0.03)	1 (0)
n=200; B=0.3; Med=10%; Ind=yes	HDMA	0.68 (0.13)	0.98 (0.01)	0.45 (0.08)	0.98 (0.01)	0.29 (0.06)	0.98 (0.01)
	HIMA	0.71 (0.13)	0.95 (0.02)	0.43 (0.08)	0.96 (0.01)	0.27 (0.06)	0.97 (0.01)
	MITM	0.58 (0.13)	1 (0)	0.32 (0.1)	1 (0)	0.17 (0.08)	1 (0)
n=500; B=0.3; Med=10%; Ind=yes	HDMA	0.72 (0.11)	0.97 (0.02)	0.89 (0.05)	0.97 (0.01)	0.75 (0.06)	0.97 (0.01)
	HIMA	0.99 (0.03)	0.98 (0.02)	0.89 (0.05)	0.97 (0.02)	0.74 (0.06)	0.97 (0.01)
	MITM	0.91 (0.06)	0.99 (0.01)	0.79 (0.06)	1 (0)	0.65 (0.07)	1 (0)
n=1000; B=0.3; Med=10%; Ind=yes	HDMA	0.33 (0.11)	0.96 (0.04)	0.98 (0.03)	0.97 (0.01)	0.95 (0.03)	0.97 (0.01)
	HIMA	1 (0)	1 (0)	0.99 (0.02)	1 (0)	0.95 (0.03)	0.99 (0.01)
	MITM	0.98 (0.03)	0.99 (0.01)	0.94 (0.04)	0.99 (0.01)	0.89 (0.04)	0.99 (0)
